# Assessment of the relationship between telomere length and atherosclerosis: A Mendelian randomization study

**DOI:** 10.1097/MD.0000000000035875

**Published:** 2023-11-17

**Authors:** Wenwen Li, Cuncheng Liu, Ying Chen, Shoupeng Dong, Menghe Zhang, Jinglong Sun, Zhenfeng Zhao, Yaoyao Zuo, Shouqiang Chen

**Affiliations:** a Second School of Clinical Medicine, Shandong University of Traditional Chinese Medicine, Jinan, China; b Department of Neonatology, Weifang City Hospital of Traditional Chinese Medicine, Weifang, China; c College of Traditional Chinese Medicine, Shandong University of Traditional Chinese Medicine, Jinan, China.

**Keywords:** atherosclerosis, cerebral atherosclerosis, coronary atherosclerosis, Mendelian randomization, peripheral atherosclerosis, telomere length

## Abstract

To evaluate the causal relationship between genetically determined telomere length (TL) and atherosclerosis (AS). We performed a 2-sample Mendelian randomization (MR) study to assess the potential causal relationship between TL and AS (coronary AS, cerebral AS, peripheral atherosclerosis (PAD), and AS, excluding cerebral, coronary, and PAD). The TL phenotype contained 472,174 participants, and the 4 subtypes of AS had 361,194, 218,792, 168,832, and 213,140 participants, all of European ancestries. The single nucleotide polymorphisms (SNPs) of TL strongly associated with the 4 atherosclerotic subtypes included in this study were 101, 92, 91, and 92, respectively. The odds ratios (ORs) and 95% confidence interval (CI) between TL and coronary AS calculated using inverse variance weighted (IVW) were 0.993 (0.988, 0.997), and the results were statistically significant (*P* < .05). The results between TL and cerebral AS, PAD, and AS (excluding cerebral, coronary, and PAD) were not statistically significant (*P* > .05). “Egger-intercept test” showed that there was no horizontal pleiotropy (*P* > .05); “leave-one-out analysis” sensitivity analysis showed that the results were stable and there were no instrumental variables with strong effects on the results; “MR- pleiotropy residual sum and outlier (PRESSO) test” showed 1 outlier for coronary AS and no outliers for the remaining subgroups. The results of the 2-sample MR analysis showed a causal association between TL and coronary AS but not with cerebral AS, PAD, and AS (excluding cerebral, coronary, and PAD). This may elucidate the observation that various vascular regions can be affected by AS but highlights the propensity of coronary arteries to be more susceptible to AS development.

## 1. Introduction

Atherosclerosis (AS) is a progressive disease that usually develops over many years, with a progression that includes early endothelial dysfunction and late vulnerable plaque formation and destruction.^[1]^ Its prevalence increases progressively with age and is the leading cause of most cardiovascular diseases (CVDs), which are the leading cause of death worldwide.^[2]^ It is often associated with other risk factors such as hypertension, diabetes mellitus, obesity, and smoking.^[3–6]^ However, the exact cause of atherosclerosis is not fully understood, and it is likely to result from a complex interplay of genetic and environmental factors. Telomeres are specialized structures located at the ends of chromosomes, and the strands along the 5’ to 3’ direction are rich in guanine (G) and thymine (T).^[7]^ Its main sequence is TTAGG and is tightly bound to many vital proteins that protect chromosome integrity and stability.^[7,8]^ In normal human cells, telomeres can gradually shorten with cell division. Telomere shortening is not simply a problem that leads to cellular senescence but has been linked to the development of several diseases, one of which is AS.^[9–14]^ Previous studies suggest that people with shorter TL may be at increased risk of atherosclerotic lesions due to the aging of vascular cells and subsequent cellular dysfunction.^[10,14–16]^ However, some studies have found no association between short telomeres and subclinical AS.^[17,18]^ Therefore, it is unclear whether telomere shortening accelerates the outcome of AS. In addition, these observational studies are often challenging to establish a causal relationship between the 2 and are susceptible to confounding factors and ethical implications.

Mendelian randomization (MR) analysis is an epidemiological method widely used in recent years to infer causal effects between exposure factors and study outcomes by using genetic variants strongly associated with exposure factors as instrumental variables.^[19–21]^ It follows Mendelian laws of inheritance, is a “natural” randomized controlled trial, and has become a hot research topic for causal inference in observational studies because of the innate nature of genetic variation and reasonable causal time sequence.^[22,23]^ Therefore, we conducted a study using the 2-sample MR method to investigate the association between TL and AS from a causal perspective.

## 2. Materials and methods

### 2.1. Study design

In this study, a 2-sample MR analysis was performed using the published genome-wide association study (GWAS) pooled dataset to assess the causal relationship between TL and AS, and sensitivity analysis was performed to test the reliability of the results.

**Figure 1.**
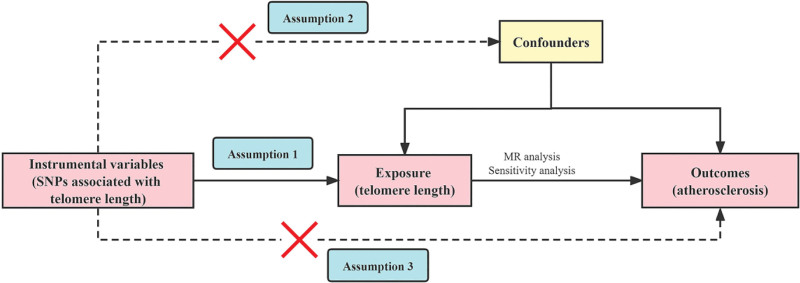
Model of the 2-sample Mendelian randomization analysis. MR = Mendelian randomization, SNPs = single nucleotide polymorphisms.

MR studies are required to satisfy 3 core assumptions.^[21]^ These include: a strong association between genetic variation and exposure factors is assumed, genetic variation is independent of confounding factors that affect “exposure and outcome,” and genetic variation can only contribute to outcome through exposure, but not through other pathways. MR assumptions are illustrated in Figure 1.

### 2.2. Data sources

The variable for exposure was TL, and the outcome variable was AS. The GWAS data for exposure and outcome were obtained from the IEU OpenGWAS project (https://gwas.mrcieu.ac.uk/) database. The former contained 472,174 European (Dataset: ieu-b-4879) and 20,134,421 single nucleotide polymorphisms (SNPs); the latter included 4 subtypes: coronary atherosclerosis (coronary AS, Dataset: ukb-d-I9_CORATHER), cerebral atherosclerosis (cerebral AS, Dataset: finn-b-I9_CEREBATHER), peripheral atherosclerosis (PAD, Dataset: finn-b-DM_PERIPHATHERO) and AS (excluding cerebral, coronary and PAD) (Dataset: finn-b-I9_ATHSCLE). In addition, the exposed and outcome data were obtained from different datasets, thus avoiding bias in causality estimation due to collinearity. Detailed information is provided in Table 1.

**Table 1:** GWAS data information of the 2-sample of Mendelian randomization study.

Trait	GWAS ID	Population	Sample size	n SNP	Yr
Telomere length	ieu-b-4879	European	472,174	20,134,421	2021
Atherosclerosis					
Coronary atherosclerosis	ukb-d-I9_CORATHER	European	361,194	13,586,589	2018
Cerebral atherosclerosis	finn-b-I9_CEREBATHER	European	218,792	16,380,466	2021
Peripheral atherosclerosis	finn-b-DM_PERIPHATHERO	European	168,832	16,380,247	2021
Atherosclerosis, excluding cerebral, coronary and PAD	finn-b-I9_ATHSCLE	European	213,140	16,380,428	2021

GWAS = genome-wide association study, PAD = peripheral atherosclerosis, SNP = single nucleotide polymorphism.

### 2.3. Selection of instrument variables

Instrumental variables were selected according to the 3 core assumptions of the MR study. The association assumption (*P* < 5 × 10^−8^) was completed using the TwoSampleMR package of R software, and the interference of linkage disequilibrium (LD) was excluded with parameters set to r^2^ = 0.001 and kb = 10,000. To satisfy core assumptions 2 and 3, we excluded SNPs associated with confounders and outcomes, that is, the SNPs we included were neither associated with confounders nor with study outcomes. By reading the literature^[3–7,13,24]^ and considering them together, we included hypertension, diabetes mellitus, obesity, dyslipidemia, smoking, and physical inactivity as confounding factors. The specific screening was done through the PhenoScanner website (http://www.phenoscanner.medschl.cam.ac.uk/),^[25]^ with criteria set by default as Catalogue: Diseases & traits, *P* value: 1E-5, Proxies: None, r^2^: 0.8, Build: 37. Then, we extracted information on instrumental variables in the outcome, harmonized exposure and outcome SNP effects and removed the SNPs for incompatible alleles, being palindromic with intermediate allele frequencies.^[26]^

### 2.4. Statistical analysis

#### 2.4.1. Weak instrumental variable analysis.

The presence of weak instrumental variable bias in the selected instrumental variables was assessed by calculating the *F* statistic, and *F* > 10 indicates the absence of weak instrumental variable bias, further validating the association assumption.^[27,28]^ *F* is calculated as $F=\frac{N-k-1}{k}\times \frac{R^{2}}{1-R^{2}}$, where *N* is the sample size of exposure, *k* is the number of instrumental variables, and *R*^2^ is the proportion of variation in exposure factors explained by instrumental variables.^[29]^ *R*^2^ is calculated as $R^{2}=\frac{2\times EAF\times (1-EAF)\times \beta^{2}}{SD^{2}}$, where *EAF* is the effect allele frequency, *β* is the effect value of the allele, and *SD* is the standard deviation. We excluded SNPs with *F* ≤ 10 and the final obtained SNPs were used to perform MR analysis.

#### 2.4.2. Mendelian randomization analysis.

In this study, inverse variance weighted (IVW)^[30]^ was used as the main analysis method to assess the potential causal relationships between TL and coronary AS, TL and cerebral AS, TL and PAD, TL and AS (excluding cerebral, coronary and PAD). IVW is the result of combining the Wald ratios of multiple SNPs. Since IVW assumes that all SNPs are valid, even if only 1 gene variant is invalid, it will be biased. Therefore, considering the existence of different underlying assumptions for different methods,^[31]^ MR-Egger,^[32]^ weighted median,^[33]^ simple mode and weighted mode^[31]^ were used to complement the MR results and to test the robustness of the results. In addition, Cochran Q tests were performed separately for the IVW method and MR-Egger method to assess the presence of heterogeneity between instrumental variables. Random-effects IVW or fixed-effects IVW was chosen according to the presence of heterogeneity.

#### 2.4.3. Pleiotropy test.

Pleiotropy is common in genetic variants, so it is difficult to exclude it completely. The Egger-intercept test is now commonly used to analyze whether there is pleiotropy among IVs. When Egger-intercept is close to 0, it indicates the absence of horizontal pleiotropy; conversely, it indicates the presence of horizontal pleiotropy.^[34,35]^ Horizontal pleiotropy can distort MR tests, leading to inaccurate causal estimates and potential false positive causality.^[36]^ Therefore, we should avoid the presence of horizontal pleiotropy whenever possible.

#### 2.4.4. Sensitivity analysis.

We used “leave-one-out analysis” to perform sensitivity analysis on the results, that is, to calculate the combined effect of the remaining SNPs after eliminating each SNP one by one. If the MR results of the analysis of the remaining SNPs after eliminating a SNP are less different from the total results, it means that the MR analysis results are robust; if the MR results are very different from the total results, it means that the MR results are sensitive to that SNP. And we used the MR - pleiotropy residual sum and outlier (PRESSO) method to detect and correct for horizontal pleiotropy outliers in MR.^[36]^

All analyses were performed using the “TwoSampleMR” (version 0.5.6) and “MR-PRESSO” packages in R software (version 4.2.1).

### 2.5. Ethical approval

The requirement for ethical review and informed consent were waived because the study analyzed public databases.

## 3. Results

### 3.1. Selection of instrument variables

There were 154 SNPs associated with TL (Supplementary Table S1, http://links.lww.com/MD/K603), excluding those with F ≤ 10, leaving 116 SNPs. 116 SNPs were extracted in the endings coronary AS, cerebral AS, PAD, and AS (excluding cerebral, coronary, and PAD), respectively. The information was obtained for 112, 103, 103, and 103 SNPs. After removing the SNPs for incompatible alleles, being palindromic with intermediate allele frequencies, SNPs associated with confounding factors and outcomes, 101, 91, 91, and 91 SNPs, were finally obtained for MR analysis, respectively, as shown in Table 2.

**Table 2:** Evaluation of Mendelian randomization estimates and reliability between telomere length and atherosclerosis.

Outcome	n SNP	Method	Cochran Q (*P*)	Beta	SE	*P*	OR (95% CI)	Egger-intercept (*P*)
Coronary AS	101	MR Egger	148.974 (<0.001)	−0.011	0.004	0.012	0.989 (0.981, 0.997)	0.0001 (.255)
		Weighted median	-	−0.008	0.003	0.013	0.992 (0.986, 0.998)	
		Inverse variance weighted	150.951 (<0.001)	−0.007	0.002	0.003	0.993 (0.988, 0.998)	
		Simple mode	-	−0.007	0.007	0.330	0.993 (0.979, 1.007)	
		Weighted mode	-	−0.010	0.004	0.011	0.990 (0.983, 0.998)	
Cerebral AS	91	MR Egger	89.170 (0.475)	−1.020	1.125	0.367	0.361 (0.040, 3.273)	0.0068 (.824)
		Weighted median	-	−1.249	1.044	0.232	0.287 (0.037, 2.219)	
		Inverse variance weighted	89.220 (0.503)	−0.810	0.616	0.189	0.445 (0.133, 1.489)	
		Simple mode	-	−1.775	2.043	0.387	0.169 (0.003, 9.298)	
		Weighted mode	-	−1.433	1.037	0.171	0.239 (0.031, 1.822)	
PAD	91	MR Egger	94.465 (0.326)	−0.186	0.169	0.274	0.830 (0.596, 1.156)	0.0054 (.244)
		Weighted median	-	−0.127	0.151	0.397	0.880 (0.655, 1.183)	
		Inverse variance weighted	95.924 (0.315)	−0.020	0.090	0.822	0.980 (0.821, 1.169)	
		Simple mode	-	−0.144	0.254	0.572	0.866 (0.526, 1.425)	
		Weighted mode	-	−0.144	0.147	0.329	0.866 (0.649, 1.155)	
AS, excluding cerebral, coronary and PAD	91	MR Egger	101.811 (0.167)	−0.131	0.165	0.430	0.877 (0.634, 1.213)	0.0039 (.386)
		Weighted median	-	−0.174	0.149	0.242	0.840 (0.628, 1.124)	
		Inverse variance weighted	102.681 (0.170)	−0.011	0.085	0.890	0.989 (0.838, 1.168)	
		Simple mode	-	0.265	0.314	0.401	1.303 (0.704, 2.413)	
		Weighted mode	-	−0.152	0.155	0.329	0.859 (0.633, 1.164)	

AS = atherosclerosis, CI = confidence interval, MR = Mendelian randomization, OR = odds ratio, PAD = peripheral atherosclerosis, SE = standard error, SNP = single nucleotide polymorphism.

### 3.2. MR analysis

This study used the IVW method as the primary analysis method, and 4 other MR analysis methods were used as supplements. The results showed a causal relationship between TL and coronary AS (IVW: OR = 0.993, 95% CI: 0.988–0.998, *P* < .01). There was no causal relationship between TL and cerebral AS (IVW: OR = 0.445, 95% CI: 0.133–1.489, *P* > .05), TL-PAD (IVW: OR = 0.980, 95% CI: 0.821–1.169, *P* > .05) and TL-AS, excluding cerebral, coronary and PAD (IVW: OR = 0.989, 95% CI: 0.838–1.168, *P* > .05). Cochran Q test showed no heterogeneity in all subtypes except for the coronary atherosclerosis subtype. The specific analysis results of the 5 methods are shown in Table 2. Visualization of the MR results is shown in the supplemental material (Supplementary Figure S1, http://links.lww.com/MD/K602).

### 3.3. Pleiotropy test

By Pleiotropy test, the Egger-intercept of TL-Coronary AS, TL-Cerebral AS, TL-PAD and TL-AS (excluding cerebral, coronary and PAD) were calculated as 0.0001, 0.0068, 0.0054 and 0.0039, respectively. All were close to 0, *P* > .05, indicating that the results of the causal effect analysis were less likely to be influenced by gene pleiotropy, as shown in Table 2.

### 3.4. Sensitivity analysis

The “leave-one-out analysis” results showed that after eliminating each SNP in turn, no significant effect of SNPs on the causal association estimates was found. The funnel plot indicates that SNP is distributed roughly symmetrically on both sides of the IVW line, indicating that causal associations are unlikely to be affected by potential bias. All the visualization results are shown in the supplemental material (Supplementary Figure S1, http://links.lww.com/MD/K602). The MR-PRESSO test showed the presence of an outlier in TL-Coronary AS, which was excluded and re-run for MR analysis, heterogeneity test and pleiotropy test. The results were not significantly changed from before, and the results are shown in Table 3. There were no outliers in TL-Cerebral AS, TL-PAD and TL-AS (excluding cerebral, coronary and PAD).

**Table 3:** Evaluation of Mendelian randomization estimates and reliability between telomere length and coronary atherosclerosis (After removing the outliers).

Outcome	n SNP	Method	Cochran Q (*P*)	Beta	SE	*P*	OR (95% CI)	Egger-intercept (*P*)
Coronary AS	100	MR Egger	140.026 (0.003)	−0.010	0.004	0.017	0.990 (0.981, 0.998)	0.0001 (.391)
		Weighted median	-	−0.008	0.003	0.013	0.992 (0.985, 0.998)	
		Inverse variance weighted	141.089 (0.004)	−0.007	0.002	0.002	0.993 (0.988, 0.997)	
		Simple mode	-	−0.007	0.007	0.367	0.993 (0.979, 1.008)	
		Weighted mode	-	−0.010	0.003	0.005	0.990 (0.984, 0.997)	

AS = atherosclerosis, CI = confidence interval, MR = Mendelian randomization, OR = odds ratio, SE = standard error, SNP = single nucleotide polymorphism.

## 4. Discussion

AS is the cause of most CVDs, and identifying the causes of AS is important for preventing, diagnosing and treating CVDs. Studying telomeres as a potential tool for the early detection of atherosclerotic disease seems to be a promising approach. Several researchers are trying to detect the exact role of TL in the progression of AS, but such investigations are very challenging. So far, the future of this field is rather vague, as the results of many studies are inconsistent.^[37]^ De Meyer et al used telomere restriction fragment analysis to measure peripheral blood leukocyte TL in 2509 volunteers aged 35–55 years and ultrasonography to determine the intima-media thickness and plaque presence in the left and right carotid and femoral arteries. The results showed no statistically significant association between telomere shortening and preclinical atherosclerosis.^[17]^ In a cross-sectional study in a middle-aged population, Fernández-Alvira et al investigated the association between leukocyte telomere length (LTL) and subclinical atherosclerosis. It was found that average LTL and short telomere load were not important independent determinants of subclinical AS.^[18]^ However, some studies have yielded different results. Nzietchueng et al assessed TL in vascular tissues to determine whether there was in vivo regulation of TL by atherosclerotic lesions. It was found that TL was longer in arterial segments that did not develop AS, whereas TL was shorter in aortic tissues that developed atherosclerotic lesions.^[38]^ Another 40-year longitudinal study suggests that people born with shorter TL may be more likely to develop atherosclerotic lesions in midlife.^[15]^ Because most of these studies are based on observational epidemiological methods, they are often used to assess the etiology of a disease without obtaining a definitive causal relationship and are susceptible to confounding factors and reverse causal associations.^[39]^ The MR method is equivalent to a “natural” randomized controlled trial, with a proper temporal order of cause and effect, which can avoid the interference of these factors and obtain more reliable results. Furthermore, the studies above did not differentiate how TL impacts the occurrence of AS in different vascular sites. To our knowledge, this study represents the first exploration of the causal relationship between TL and AS in distinct vascular sites through MR methodology.

This study used MR analysis to investigate the association between TL and AS. Among these, the AS phenotype encompasses 4 distinct subtypes: coronary AS, cerebral AS, PAD, and AS (excluding cerebral, coronary and PAD). MR findings suggested a causal association between TL and coronary AS, with longer TL associated with a lower risk of coronary AS and shorter TL associated with a higher risk of coronary AS. Apart from the causal association identified between TL and coronary AS, no causal relationship was observed between TL and cerebral AS, PAD, and AS (excluding cerebral, coronary, and PAD). This may elucidate the inconsistencies observed in various research outcomes, suggesting that the causal relationships between TL and AS in distinct vascular sites could vary.

Telomeres are widely regarded as the biological clock of human existence, intricately linked to aging. AS is a vascular disorder that can impact arteries throughout the body. Although various vascular sites may be susceptible, certain vessels are predisposed to atherosclerosis more readily. Our study potentially elucidates the reason behind the greater vulnerability of coronary arteries to AS with the shortening of telomeres and the advancement of age, consequently leading to a spectrum of CVDs that imperil human health. Previous studies have shown that telomere shortening leads to aging and damage of coronary vascular endothelial cells, thereby rendering the vessel wall vulnerable to injury and consequent AS.^[40]^ The research conducted by Mainous, Arch G 3rd et al, examining the relationship between TL and coronary artery calcification in a sample of 325 middle-aged individuals without a history of CVDs, similarly confirms an inverse correlation between TL and the presence of coronary AS.^[41]^ Our findings are consistent with them. Furthermore, the impact of coronary AS on the coronary arteries gives rise to the manifestation of coronary artery disease (CAD). Numerous studies have reported the association between TL and CAD. The research conducted by D Nose et al indicates a concordant trend between the severity of CAD and the shortening of telomere G-tail length. Patients exhibiting telomere G-tail length shortening are at a heightened risk of cardiovascular events.^[42]^ M Rafiq et al, through a case-control study with meta-analysis, demonstrated an association between LTL and the occurrence of CAD, suggesting its potential utility as a diagnostic and predictive marker for identifying CAD patients.^[43]^ The research conducted by M Xiang et al indicates a correlation between LTL and reduced incidence of coronary heart disease.^[44]^

The horizontal pleiotropy test was insignificant, indicating that other confounding factors less influenced the instrumental variables we used and that the findings were robust. The leave-one-out test showed no significant change in the observed effect values after removing individual SNPs one by one, and a negative correlation between TL and coronary AS was still observed, with no causal relationship between TL and brain AS and PAD and AS (excluding brain, coronary and PAD), indicating stable results. This study avoids the problems of reverse causality associations and potential confounders in observational studies. It is not required to suffer from the limitations of expensive, time-consuming, and ethical issues of randomized control trials. However, the study has some limitations. First, the genetic variants used in the study inevitably have the problem of pleiotropy, which cannot be completely ruled out and can only minimize the bias caused by pleiotropy on the conclusions. Second, this study used a population sample of European ancestry, and the lack of data from other ethnic groups limits the generalizability of the findings. Data analysis from other ethnic groups is needed to make the results more reliable. Finally, since the 2-sample MR was based on a large sample design, a larger sample of exposure and outcome could have been obtained to validate the results’ reliability further.

## 5. Conclusion

In conclusion, this study investigated the causal relationship between TL and AS using a 2-sample MR method. The results showed a causal association between TL and coronary AS. Greater TL is associated with a reduced risk of coronary AS, while shorter TL is associated with an elevated risk of coronary AS. And there was no causal association with cerebral AS, PAD, and AS (excluding cerebral, coronary, and PAD). This may elucidate the observation that various vascular regions can be affected by AS but highlights the propensity of coronary arteries to be more susceptible to AS development.

## Acknowledgments

We thank the IEU Open GWAS database for providing the data set.

## Author contributions

**Conceptualization:** Wenwen Li, Shouqiang Chen.

**Data curation:** Cuncheng Liu, Ying Chen.

**Funding acquisition:** Yaoyao Zuo, Shouqiang Chen.

**Investigation:** Zhenfeng Zhao.

**Project administration:** Menghe Zhang, Jinglong Sun.

**Software:** Shoupeng Dong.

**Writing – original draft:** Wenwen Li, Cuncheng Liu.

**Writing – review & editing:** Wenwen Li, Shouqiang Chen.

## Supplementary Material





## References

[1] Tedgui A Mallat Z. Cytokines in atherosclerosis: pathogenic and regulatory pathways. Physiol Rev. 2006;86:515–81. 16601268 10.1152/physrev.00024.2005

[2] Libby P Buring JE Badimon L. Atherosclerosis. Nat Rev Dis Primers. 2019;5:56. 31420554 10.1038/s41572-019-0106-z

[3] Tegos TJ Kalodiki E Sabetai MM. The genesis of atherosclerosis and risk factors: a review. Angiology. 2001;52:89–98. 11228092 10.1177/000331970105200201

[4] Glasser SP Selwyn AP Atherosclerosis GP. Risk factors and the vascular endothelium. Am Heart J. 1996;131:379–84. 8579037 10.1016/s0002-8703(96)90370-1

[5] Libby P. The changing landscape of atherosclerosis. Nature. 2021;592:524–33. 33883728 10.1038/s41586-021-03392-8

[6] Falk E. Pathogenesis of atherosclerosis. J Am Coll Cardiol. 2006;47:C7–12. 16631513 10.1016/j.jacc.2005.09.068

[7] Fyhrquist F Saijonmaa O. Telomere length and cardiovascular aging. Ann Med. 2012;44(sup1):S138–42. 22713142 10.3109/07853890.2012.660497

[8] Doksani Y. The response to DNA damage at telomeric repeats and its consequences for telomere function. Genes. 2019;10:318. 31022960 10.3390/genes10040318 PMC6523756

[9] Schneider CV Schneider KM Teumer A. Association of Telomere Length With Risk of Disease and Mortality. JAMA Intern Med. 2022;182:291–300. 35040871 10.1001/jamainternmed.2021.7804 PMC8767489

[10] Yegorov YE Poznyak AV Nikiforov NG. Role of telomeres shortening in atherogenesis: an overview. Cells. 2021;10:395. 33671887 10.3390/cells10020395 PMC7918954

[11] Boniewska-Bernacka E Pańczyszyn A Klinger M. Telomeres and telomerase in risk assessment of cardiovascular diseases. Exp Cell Res. 2020;397:112361. 33171154 10.1016/j.yexcr.2020.112361

[12] Hoffmann J Richardson G Haendeler J. Telomerase as a therapeutic target in cardiovascular disease. Arterioscler Thromb Vasc Biol. 2021;41:1047–61. 33504179 10.1161/ATVBAHA.120.315695

[13] Tamura Y Takubo K Aida J. Telomere attrition and diabetes mellitus. Geriatr Gerontol Int. 2016;16(Suppl 1):66–74. 27018285 10.1111/ggi.12738

[14] Yin H Pickering JG. Telomere length: implications for atherogenesis. Curr Atheroscler Rep. 2023;25:95–103. 36689071 10.1007/s11883-023-01082-6 PMC9947063

[15] Niu Z Wen X Buka SL. Associations of telomere length at birth with predicted atherosclerotic lesions and cardiovascular disease risk factors in midlife: a 40-year longitudinal study. Atherosclerosis. 2021;333:67–74. 34428605 10.1016/j.atherosclerosis.2021.08.013

[16] Samani NJ Boultby R Butler R. Telomere shortening in atherosclerosis. Lancet. 2001;358:472–3. 11513915 10.1016/S0140-6736(01)05633-1

[17] De Meyer T Rietzschel ER De Buyzere ML. Systemic telomere length and preclinical atherosclerosis: the Asklepios Study. Eur Heart J. 2009;30:3074–81. 19687155 10.1093/eurheartj/ehp324

[18] Fernández-Alvira JM Fuster V Dorado B. Short telomere load, telomere length, and subclinical atherosclerosis: the PESA study. J Am Coll Cardiol. 2016;67:2467–76. 27230041 10.1016/j.jacc.2016.03.530

[19] Gagliano Taliun SA Evans DM. Ten simple rules for conducting a mendelian randomization study. PLoS Comput Biol. 2021;17:e1009238. 34383747 10.1371/journal.pcbi.1009238 PMC8360373

[20] Lawlor DA Harbord RM Sterne JAC. Mendelian randomization: using genes as instruments for making causal inferences in epidemiology. Statist Med. 2008;27:1133–63. 10.1002/sim.3034 17886233

[21] Emdin CA Khera AV Kathiresan S. Mendelian randomization. JAMA. 2017;318:1925–6. 29164242 10.1001/jama.2017.17219

[22] Ellervik C Roselli C Christophersen IE. Assessment of the relationship between genetic determinants of thyroid function and atrial fibrillation: a Mendelian randomization study. JAMA Cardiol. 2019;4:144–52. 30673084 10.1001/jamacardio.2018.4635 PMC6396813

[23] Khasawneh LQ Al-Mahayri ZN Ali BR. Mendelian randomization in pharmacogenomics: the unforeseen potentials. Biomed Pharmacother. 2022;150:112952. 35429744 10.1016/j.biopha.2022.112952

[24] Tellechea ML Pirola CJ. The impact of hypertension on leukocyte telomere length: a systematic review and meta-analysis of human studies. J Hum Hypertens. 2017;31:99–105. 27357526 10.1038/jhh.2016.45

[25] Staley JR Blackshaw J Kamat MA. PhenoScanner: a database of human genotype-phenotype associations. Bioinformatics. 2016;32:3207–9. 27318201 10.1093/bioinformatics/btw373 PMC5048068

[26] Hartwig FP Davies NM Hemani G. Two-sample Mendelian randomization: avoiding the downsides of a powerful, widely applicable but potentially fallible technique. Int J Epidemiol. 2016;45:1717–26. 28338968 10.1093/ije/dyx028 PMC5722032

[27] Burgess S Thompson SG. Bias in causal estimates from Mendelian randomization studies with weak instruments. Stat Med. 2011;30:1312–23. 21432888 10.1002/sim.4197

[28] Burgess S Thompson SG, CRP CHD Genetics Collaboration. Avoiding bias from weak instruments in Mendelian randomization studies. Int J Epidemiol. 2011;40:755–64. 21414999 10.1093/ije/dyr036

[29] Pierce BL Ahsan H Vanderweele TJ. Power and instrument strength requirements for Mendelian randomization studies using multiple genetic variants. Int J Epidemiol. 2011;40:740–52. 20813862 10.1093/ije/dyq151 PMC3147064

[30] Burgess S Butterworth A Thompson SG. Mendelian randomization analysis with multiple genetic variants using summarized data. Genet Epidemiol. 2013;37:658–65. 24114802 10.1002/gepi.21758 PMC4377079

[31] Hartwig FP Davey Smith G Bowden J. Robust inference in summary data Mendelian randomization via the zero modal pleiotropy assumption. Int J Epidemiol. 2017;46:1985–98. 29040600 10.1093/ije/dyx102 PMC5837715

[32] Bowden J Davey Smith G Burgess S. Mendelian randomization with invalid instruments: effect estimation and bias detection through Egger regression. Int J Epidemiol. 2015;44:512–25. 26050253 10.1093/ije/dyv080 PMC4469799

[33] Bowden J Davey Smith G Haycock PC. Consistent estimation in Mendelian randomization with some invalid instruments using a weighted median estimator. Genet Epidemiol. 2016;40:304–14. 27061298 10.1002/gepi.21965 PMC4849733

[34] Yavorska OO Burgess S. MendelianRandomization: an R package for performing Mendelian randomization analyses using summarized data. Int J Epidemiol. 2017;46:1734–9. 28398548 10.1093/ije/dyx034 PMC5510723

[35] Burgess S Thompson SG. Interpreting findings from Mendelian randomization using the MR-Egger method. Eur J Epidemiol. 2017;32:377–89. 28527048 10.1007/s10654-017-0255-x PMC5506233

[36] Verbanck M Chen CY Neale B. Detection of widespread horizontal pleiotropy in causal relationships inferred from Mendelian randomization between complex traits and diseases. Nat Genet. 2018;50:693–8. 29686387 10.1038/s41588-018-0099-7 PMC6083837

[37] Sagris M Theofilis P Antonopoulos AS. Telomere length: a cardiovascular biomarker and a novel therapeutic target. Int J Mol Sci . 2022;23:16010. 36555658 10.3390/ijms232416010 PMC9781338

[38] NzietchuengRElfarraMNlogaJ. Telomere length in vascular tissues from patients with atherosclerotic disease. J Nutr Health Aging. 2011;15:153–6.2136517010.1007/s12603-011-0029-1

[39] Andrews SJ Goate A Anstey KJ. Association between alcohol consumption and Alzheimer’s disease: a Mendelian randomization study. Alzheimer's Dementia. 2020;16:345–53. 10.1016/j.jalz.2019.09.086 PMC7057166 31786126

[40] Ogami M Ikura Y Ohsawa M. Telomere shortening in human coronary artery diseases. Arterioscler Thromb Vasc Biol. 2004;24:546–50. 14726417 10.1161/01.ATV.0000117200.46938.e7

[41] Mainous AG Codd V Diaz VA. Leukocyte telomere length and coronary artery calcification. Atherosclerosis. 2010;210:262–7. 19945703 10.1016/j.atherosclerosis.2009.10.047

[42] Nose D Shiga Y Takahashi RU. Association between telomere G-tail length and coronary artery disease or statin treatment in patients with cardiovascular risks - a cross-sectional study. Circ Rep. 2023;5:338–47. 37564879 10.1253/circrep.CR-23-0038 PMC10411992

[43] Rafiq M Liaquat A Javed A. Association of leukocyte telomere attrition in coronary artery disease in Pakistani population: a case-control study with meta-analysis. Clin Chim Acta. 2023;547:117416. 37276942 10.1016/j.cca.2023.117416

[44] Xiang M Pilling LC Melzer D. Does physical activity moderate the association between shorter leukocyte telomere length and incident coronary heart disease? Data from 54,180 UK Biobank participants. Geroscience. Published online August 7, 2023. 10.1007/s11357-023-00890-7 PMC10828302 37544968

