# Supplementary material for: Assessment of the relationship between telomere length and atherosclerosis: A Mendelian randomization study
Source: Medicine (Baltimore). 2023 Nov 17;102(46):e35875. doi: 10.1097/MD.0000000000035875 (PMC10659599; doi:10.1097/MD.0000000000035875)
Supplement: Supplementary file 1 [file medi-102-e35875-s001.pdf]

Table S1. Information on SNPs for instrumental variable

SNP	Chr	pos	EA	OA	Exposure			Outcome			R^2	F
					Beta	SE	pval	Beta	SE	pval		
**TL-Coronary AS**												
rs1003322	22	51072289	A	C	0.0141734	0.00247546	1.00E-08	-0.000666105	0.000557982	0.232567	2.33356E-05	11.0
rs10112752	8	73958718	A	G	-0.0287522	0.00202518	9.50E-46	5.32E-05	0.000455824	0.907085	0.000209304	98.8
rs1023767	8	95530969	A	G	-0.0183732	0.00234772	5.00E-15	3.85E-05	0.000531897	0.942289	4.69924E-05	22.2
rs10768683	11	5247791	G	C	0.0469922	0.00277015	1.50E-64	-0.000402891	0.00063166	0.523585	0.000162964	77.0
rs10773176	12	122944713	G	A	-0.0172009	0.00228534	5.20E-14	0.000414455	0.000516838	0.422609	4.6027E-05	21.7
rs10805346	4	9920347	C	T	0.0117072	0.00202147	7.00E-09	0.000127672	0.000456159	0.779566	3.49945E-05	16.5
rs10840270	11	9629553	G	C	0.014383	0.00212494	1.30E-11	0.000111496	0.000481163	0.816754	4.38112E-05	20.7
rs10845387	12	11757743	A	G	-0.0141214	0.00209396	1.50E-11	9.94E-05	0.000473139	0.833586	4.39783E-05	20.8
rs10905255	10	5870267	T	G	-0.0182493	0.00203099	2.60E-19	-2.60E-05	0.000459128	0.954843	8.33512E-05	39.4
rs11085072	19	4368142	T	C	-0.0131806	0.00236713	2.60E-08	-4.18E-07	0.000527928	0.999369	2.37417E-05	11.2
rs11117354	16	88092092	C	T	0.0232506	0.00219601	3.40E-26	-0.000179615	0.000499788	0.719308	0.000100368	47.4
rs111527438	17	29252703	C	T	0.0125	0.00211016	3.10E-09	-2.01E-05	0.000469635	0.965812	3.38697E-05	16.0
rs111950327	16	48283993	C	G	0.0238271	0.00409406	5.90E-09	1.81E-06	0.000919452	0.998429	8.54788E-06	4.0
rs11212631	11	108304509	C	T	-0.0193458	0.00256554	4.70E-14	-0.00102785	0.000573068	0.0728786	3.84242E-05	18.1
rs112394943	3	197842892	C	T	-0.0198961	0.00281641	1.60E-12	0.000391242	0.000643056	0.542916	2.88024E-05	13.6
rs113525195	14	23499321	A	C	-0.0124075	0.00224132	3.10E-08	-3.64E-05	0.000505933	0.942709	2.67405E-05	12.6
rs11412296	15	50366116	T	TA	0.0332259	0.00234478	1.40E-45	0.000935561	0.000527203	0.0759696	0.000155404	73.4
rs11426156	3	128318179	T	TA	-0.0115882	0.0020709	2.20E-08	0.000791032	0.000468456	0.0912977	3.18186E-05	15.0
rs11557154	9	34107505	T	C	-0.0343719	0.00298538	1.10E-30	-0.000619606	0.00067691	0.360012	6.35161E-05	30.0
rs11579626	1	146741960	C	A	0.0265113	0.00357752	1.30E-13	-0.000835465	0.000810489	0.302628	1.80684E-05	8.5
rs11584821	1	114419489	T	C	-0.0306517	0.00263623	3.00E-31	-0.000208205	0.000589856	0.724107	8.31216E-05	39.3
rs116863223	18	709396	A	G	-0.0817874	0.00937157	2.60E-18	0.00293361	0.00204285	0.150992	3.75021E-06	1.8
rs11699829	20	62157200	A	G	0.0641957	0.00602028	1.50E-26	-0.000303192	0.00131361	0.817465	1.58839E-05	7.5
rs117034449	10	103855348	A	G	0.0374377	0.00667898	2.10E-08	0.00263788	0.00147271	0.0732673	3.02912E-06	1.4
rs117407747	7	159117178	T	C	0.0450533	0.00611706	1.80E-13	0.00231521	0.00134555	0.0853179	6.16036E-06	2.9
rs117512405	20	62574274	A	G	-0.0790134	0.00824611	9.50E-22	-0.00146683	0.00181788	0.41973	6.51496E-06	3.1
rs117630647	7	124779510	A	G	0.059565	0.00720413	1.40E-16	0.00180539	0.00158563	0.254875	6.04913E-06	2.9
rs11769630	7	50257703	A	T	-0.0256807	0.00389475	4.30E-11	-0.000308985	0.00086418	0.720683	1.23402E-05	5.8
rs11991877	8	56664524	A	T	-0.030138	0.00318686	3.20E-21	-0.000272462	0.000728223	0.708296	3.72903E-05	17.6
rs12369950	12	24762109	C	T	-0.0178308	0.00290205	8.00E-10	-0.000875168	0.000659702	0.184638	1.93301E-05	9.1
rs12412214	10	101276256	A	G	-0.0245174	0.00222685	3.40E-28	-1.45E-05	0.000500193	0.97692	0.000103458	48.9
rs12451892	17	2247982	C	T	-0.0116145	0.00207578	2.20E-08	7.06E-05	0.000467886	0.880057	3.12584E-05	14.8
rs1291143	20	35525640	C	A	0.0493145	0.0027991	1.80E-69	-0.00106994	0.000638324	0.0937044	0.000168525	79.6
rs12925933	16	90141355	C	A	-0.0146622	0.00213796	7.00E-12	0.000654054	0.000481878	0.174687	4.45625E-05	21.0
rs12932179	16	9072085	G	A	-0.0136257	0.0020276	1.80E-11	9.06E-05	0.000457327	0.842947	4.71001E-05	22.2
rs13062095	3	101267385	C	T	0.0138552	0.00214113	9.70E-11	-0.000304811	0.000478531	0.524143	3.90845E-05	18.5
rs131797	22	50971631	T	TAAAAA	0.0243752	0.00236561	6.80E-25	-0.000834804	0.000531588	0.116324	8.09964E-05	38.2

rs13230646	7	23930316	C	T	-0.0173277	0.00232377	8.90E-14	-0.000151726	0.00052402	0.772167	4.40351E-05	20.8
rs1332941	13	41695100	G	A	0.0256552	0.00273159	5.90E-21	-0.000347416	0.000620207	0.57537	5.5037E-05	26.0
rs137901416	14	73418095	A	G	0.04572	0.00332355	4.70E-43	0.000240967	0.000744583	0.74622	7.23397E-05	34.2
rs139669835	18	729871	T	C	-0.0612563	0.0105346	6.10E-09	0.00169093	0.00245575	0.491101	1.32866E-06	0.6
rs139795227	1	92842367	C	A	0.0599379	0.00873247	6.70E-12	0.000583809	0.00190103	0.758766	2.75869E-06	1.3
rs141214782	5	78954683	TTATC	T	-0.0246687	0.00335723	2.00E-13	0.000989225	0.000777872	0.203478	2.08029E-05	9.8
rs142426306	20	62488152	T	C	-0.0504903	0.00539933	8.70E-21	-0.002035	0.00120029	0.0899953	1.40677E-05	6.6
rs142730696	6	26360443	TTTTTC	T	0.0216931	0.00301569	6.30E-13	0.000888805	0.000668538	0.183692	2.57411E-05	12.2
rs143190905	20	62291767	T	G	-0.0723995	0.00369421	1.60E-85	-0.00123055	0.000857142	0.151104	0.00012029	56.8
rs144204502	17	76183233	T	C	-0.100574	0.00913369	3.40E-28	0.000152377	0.00206148	0.941077	6.37053E-06	3.0
rs145114957	1	94322469	G	C	0.0272605	0.00498872	4.60E-08	-0.00137732	0.00116664	0.237765	5.16402E-06	2.4
rs150150565	18	708207	T	C	0.063762	0.00739877	6.80E-18	0.000446043	0.0016405	0.785702	6.60453E-06	3.1
rs1611236	6	29748690	A	G	-0.0160135	0.00213359	6.10E-14	2.21E-05	0.000483299	0.963473	5.24992E-05	24.8
rs16978028	18	42070981	T	A	-0.029945	0.00285068	8.20E-26	0.000459575	0.000646522	0.477183	5.75215E-05	27.2
rs17445108	12	57082058	A	G	-0.0168922	0.00300983	2.00E-08	-0.000129056	0.00066432	0.845966	1.47873E-05	7.0
rs17677991	15	42032383	G	C	0.0222664	0.00210806	4.40E-26	-0.000164663	0.000474141	0.728375	0.000106363	50.2
rs17803849	2	210673445	T	C	0.0273203	0.00203482	4.20E-41	0.000618096	0.000460781	0.179789	0.000184024	86.9
rs182059586	16	14652220	C	T	-0.0571159	0.00680853	4.90E-17	-0.00207936	0.0015006	0.165843	7.29689E-06	3.4
rs185174247	5	138914024	A	G	0.0372806	0.00435145	1.10E-17	-0.0015103	0.00100096	0.131337	1.64605E-05	7.8
rs188918174	2	54473646	T	C	0.0403062	0.00543604	1.20E-13	0.00109648	0.00118799	0.356025	8.10429E-06	3.8
rs1907702	12	88955469	A	G	0.0150247	0.00242651	5.90E-10	-0.00069966	0.000546143	0.200162	2.90418E-05	13.7
rs1957937	14	96181360	T	A	0.0209365	0.00273361	1.90E-14	0.000323963	0.000623677	0.603454	3.34239E-05	15.8
rs1985369	7	159119220	G	A	-0.0311893	0.00300952	3.60E-25	0.000267628	0.000681082	0.69436	5.20645E-05	24.6
rs202034370	2	54488018	T	TA	0.102784	0.00650108	2.60E-56	-0.000736235	0.00145186	0.612085	2.53953E-05	12.0
rs2056726	7	99780283	A	G	-0.0228078	0.00243638	7.90E-21	-0.00086761	0.000546001	0.112055	6.25166E-05	29.5
rs2230590	3	49936102	C	T	-0.0158022	0.00200806	3.60E-15	0.000857182	0.00045143	0.0575891	6.55456E-05	31.0
rs2276182	18	51798047	G	C	0.0233529	0.00204247	2.80E-30	-0.00129297	0.000460531	0.00499195	0.000133247	62.9
rs2282764	4	2255063	G	A	-0.0224234	0.00289392	9.30E-15	0.000680703	0.000669123	0.309008	3.10536E-05	14.7
rs2538745	7	76310784	C	T	-0.012942	0.002056	3.10E-10	-0.00135448	0.000460615	0.0032762	4.01839E-05	19.0
rs2555104	2	17841243	C	A	-0.0139717	0.00203498	6.60E-12	-0.00118262	0.000455687	0.00945235	4.90537E-05	23.2
rs28363070	5	1415068	A	G	0.0755557	0.00959987	3.50E-15	-0.00199879	0.00211861	0.345454	3.46598E-06	1.6
rs28502153	22	17469049	A	C	-0.0215916	0.00206208	1.20E-25	0.000179836	0.000464611	0.698705	0.000109182	51.6
rs28577594	12	123895906	C	G	0.0187657	0.00224024	5.40E-17	0.000796318	0.000506096	0.115615	6.12176E-05	28.9
rs2967355	16	82200103	C	A	-0.0461595	0.00238972	4.00E-83	0.00164397	0.000534373	0.00209503	0.000276204	130.5
rs2977608	1	768253	C	A	0.0129483	0.00233716	3.00E-08	-0.00015683	0.000531756	0.768049	2.47654E-05	11.7
rs3093888	14	20812951	A	G	-0.028973	0.00452459	1.50E-10	-0.000166252	0.00103018	0.871794	8.4544E-06	4.0
rs34550383	14	91971787	C	CT	-0.0192236	0.00200412	8.60E-22	-0.000169389	0.000452612	0.70822	9.65538E-05	45.6
rs35446936	3	169486508	A	G	-0.0940025	0.00232918	1.00E-200	0.00106854	0.000524515	0.0416303	0.001271507	601.1
rs376641875	2	54622978	C	CATAA	-0.0265863	0.00422973	3.30E-10	0.00102442	0.000935331	0.273406	1.10563E-05	5.2
rs3767952	1	41231032	A	G	0.0134472	0.00238826	1.80E-08	0.000124525	0.000540431	0.817767	2.35418E-05	11.1
rs3785074	16	69406986	G	A	0.023863	0.00220455	2.60E-27	-0.000341992	0.000498107	0.492346	0.000102118	48.2

rs3891167	18	658423	G	A	-0.0425685	0.00239551	1.20E-70	0.00019075	0.000536256	0.72206	0.000253071	119.5
rs41269079	1	45252015	A	T	0.0153617	0.0025499	1.70E-09	0.00016223	0.000571498	0.776512	2.35628E-05	11.1
rs41304832	20	62375508	A	G	0.0611702	0.0093095	5.00E-11	0.00213754	0.00222833	0.337431	2.23561E-06	1.1
rs4498805	1	110910397	T	G	0.0150601	0.00200376	5.70E-14	0.00077152	0.000453255	0.0887238	5.92977E-05	28.0
rs4530278	19	33752994	T	G	0.0138793	0.0020567	1.50E-11	0.00047648	0.00046367	0.304126	4.63655E-05	21.9
rs45604339	14	65543102	T	C	-0.020433	0.00211433	4.30E-22	0.000107275	0.0004726	0.820432	8.90747E-05	42.1
rs4616688	3	160042459	T	G	-0.0173476	0.00200198	4.50E-18	0.000177313	0.00045279	0.695354	7.93059E-05	37.4
rs4695407	4	48843372	G	A	0.0141511	0.00199925	1.50E-12	0.000335743	0.000452663	0.458265	5.30405E-05	25.0
rs4724	17	7760397	A	G	-0.0547446	0.00312441	9.80E-69	-0.000544878	0.000722849	0.450975	0.000133944	63.3
rs4731541	7	128678236	G	C	-0.0206119	0.00205962	1.40E-23	-0.000466259	0.000466109	0.317156	9.94367E-05	47.0
rs4743037	9	109639970	T	C	0.0147971	0.00238094	5.10E-10	-0.00115088	0.00054135	0.0335097	2.90507E-05	13.7
rs56061761	16	70187811	A	G	-0.0203556	0.0022296	6.90E-20	-0.000787856	0.000501487	0.116175	7.8375E-05	37.0
rs5742915	15	74336633	C	T	0.0193377	0.00202886	1.60E-21	-0.000443173	0.0004525	0.327389	9.50704E-05	44.9
rs59409453	17	1666218	G	A	0.0202133	0.00230175	1.60E-18	-0.000459157	0.00052106	0.378211	6.42926E-05	30.4
rs6007020	22	45790132	C	T	0.0144904	0.00209637	4.80E-12	0.00100829	0.00047238	0.0328035	4.70577E-05	22.2
rs6054257	20	66370	A	G	-0.0141684	0.00247729	1.10E-08	0.000137977	0.000559025	0.805049	2.27012E-05	10.7
rs611646	11	108177097	A	T	-0.0368309	0.00203547	3.50E-73	-0.000280812	0.000458965	0.540646	0.000335143	158.3
rs61748181	5	1294166	T	C	-0.059181	0.00595394	2.80E-23	0.000899888	0.00131873	0.494994	1.17558E-05	5.6
rs6536702	4	164028105	A	G	0.0534148	0.00238875	9.40E-111	-0.000922954	0.000539614	0.0871927	0.000369723	174.6
rs6584579	10	105645725	G	A	0.0114923	0.00204674	2.00E-08	-0.000747438	0.000463439	0.106787	3.20198E-05	15.1
rs6587577	1	151402045	G	A	-0.0182148	0.0026359	4.80E-12	0.00127317	0.000599882	0.0338073	2.90246E-05	13.7
rs6590343	11	128500215	G	A	0.0121739	0.00201444	1.50E-09	-0.000104792	0.000450962	0.816247	3.86324E-05	18.2
rs6659669	1	185315067	T	C	-0.0117091	0.00205167	1.10E-08	-0.000419596	0.000460342	0.362039	3.29668E-05	15.6
rs6669563	1	32279629	A	G	0.0182358	0.00202476	2.10E-19	-0.000851453	0.000454955	0.0612745	8.45649E-05	39.9
rs66731853	1	20916238	A	G	-0.0177791	0.00215421	1.50E-16	0.000338662	0.000482062	0.482351	6.24992E-05	29.5
rs6751209	2	43588302	C	T	-0.0140465	0.00248465	1.60E-08	0.000302789	0.000562659	0.590481	2.2001E-05	10.4
rs6776756	3	128215821	A	G	-0.0174439	0.00203747	1.10E-17	0.000247014	0.000458502	0.590065	7.46646E-05	35.3
rs6790988	3	170263320	G	A	0.0145728	0.00228428	1.80E-10	-0.000120765	0.000513103	0.813927	3.30101E-05	15.6
rs6881568	5	1670265	A	C	0.0169256	0.00207735	3.70E-16	-5.07E-05	0.000468104	0.913718	6.4987E-05	30.7
rs7099229	10	96134685	A	G	-0.0153288	0.00224403	8.40E-12	-0.00011331	0.000510053	0.824195	3.92539E-05	18.5
rs7164950	15	56775385	G	A	0.0129362	0.00204001	2.30E-10	-0.00124723	0.000458061	0.00647232	4.10754E-05	19.4
rs7209057	17	65705530	A	G	0.011819	0.00202865	5.70E-09	-8.65E-05	0.00045762	0.850068	3.54073E-05	16.7
rs7221585	17	76195153	T	C	0.0143271	0.00247042	6.70E-09	-0.000431779	0.000562943	0.44308	2.47643E-05	11.7
rs73581419	14	21941148	T	C	0.0229838	0.00324156	1.30E-12	-0.000895826	0.000722353	0.214921	2.02808E-05	9.6
rs73730598	5	77973	A	G	0.0273632	0.00439272	4.70E-10	-0.000700828	0.00099528	0.481339	8.51229E-06	4.0
rs76065543	16	74678063	T	C	0.0342843	0.00290708	4.20E-32	-0.00129777	0.000647668	0.0450962	6.98776E-05	33.0
rs76219171	16	50188929	A	G	0.0359839	0.00431741	7.80E-17	0.000113841	0.000957925	0.905401	1.61885E-05	7.6
rs762679	8	48885436	A	T	0.0310104	0.00285024	1.40E-27	-0.00105997	0.000639369	0.0973509	6.16249E-05	29.1
rs76666449	12	120904895	C	T	0.0295125	0.00333186	8.20E-19	-0.00270683	0.000743694	0.000272979	3.00755E-05	14.2
rs7705526	5	1285974	A	C	0.0776022	0.00216124	1.00E-200	-0.0006159	0.000487473	0.206427	0.001201003	567.8
rs77231040	10	106280527	C	G	0.0989303	0.0134649	2.00E-13	-0.000198611	0.0030472	0.948032	1.3054E-06	0.6

rs7772289	6	28674322	T	G	0.017549	0.00200003	1.70E-18	-0.00029893	0.000450987	0.507437	8.15236E-05	38.5
rs77732866	2	58979879	A	G	0.0177942	0.00290595	9.20E-10	0.000141569	0.000657894	0.829624	1.88464E-05	8.9
rs7790856	7	124459852	T	C	-0.0437199	0.00220526	1.80E-87	-0.000185849	0.000497707	0.708845	0.000342183	161.6
rs78491606	3	72891547	C	A	-0.0756311	0.00741168	1.90E-24	-0.000227262	0.00162624	0.88886	7.98016E-06	3.8
rs79977579	12	54694560	A	C	0.0281517	0.00343182	2.30E-16	0.000333626	0.000759028	0.660268	2.46322E-05	11.6
rs80116508	16	3650970	A	G	-0.0352672	0.00415151	2.00E-17	0.000192628	0.000934985	0.836773	1.7872E-05	8.4
rs80324517	6	204031	A	G	0.0396515	0.00466286	1.80E-17	-0.000882986	0.00104584	0.398512	1.40682E-05	6.6
rs8102497	19	57370055	A	G	-0.0149654	0.0020233	1.40E-13	-0.000699704	0.000456218	0.125103	5.68558E-05	26.8
rs8105767	19	22215441	G	A	0.0328384	0.00220117	2.50E-50	1.26E-05	0.00049897	0.979864	0.000195935	92.5
rs869785	3	24347800	C	T	-0.0147303	0.00212801	4.40E-12	-0.00110922	0.000481046	0.0211203	4.4702E-05	21.1
rs871134	4	7044380	T	C	-0.0182986	0.0020263	1.70E-19	-0.000163074	0.000457603	0.721566	8.47106E-05	40.0
rs932002	1	226577306	T	C	-0.0402052	0.00279667	7.30E-47	0.00085008	0.00063181	0.178475	0.00011213	53.0
rs9398196	6	109601554	G	A	-0.0143586	0.00201175	9.50E-13	0.000274627	0.00045123	0.542776	5.38574E-05	25.4
rs939916	11	202253	A	G	0.0241795	0.00216724	6.60E-29	-0.000970181	0.000489078	0.0472912	0.000116579	55.1
rs9419958	10	105675946	C	T	-0.0810098	0.00293847	2.60E-167	0.00166141	0.000670645	0.013237	0.000384375	181.6
rs9600019	13	73317585	T	C	0.0127134	0.00213096	2.40E-09	-9.87E-05	0.000477673	0.836239	3.36154E-05	15.9
rs9940099	16	3613207	T	G	-0.033609	0.00411613	3.20E-16	0.000351024	0.000919505	0.702645	1.66006E-05	7.8
rs9955360	18	78008334	A	C	-0.0190311	0.00299791	2.20E-10	-0.000314659	0.000685131	0.646042	1.93954E-05	9.2
**TL-Cerebral AS**												
rs1003322	22	51072289	A	C	0.0141734	0.00247546	1.00E-08	-0.1289	0.1969	0.5128	2.33356E-05	11.0
rs10112752	8	73958718	A	G	-0.0287522	0.00202518	9.50E-46	0.0637	0.1399	0.6486	0.000209304	98.8
rs1023767	8	95530969	A	G	-0.0183732	0.00234772	5.00E-15	-0.0952	0.1438	0.508	4.69924E-05	22.2
rs10768683	11	5247791	G	C	0.0469922	0.00277015	1.50E-64	0.1211	0.1726	0.4831	0.000162964	77.0
rs10773176	12	122944713	G	A	-0.0172009	0.00228534	5.20E-14	0.0989	0.1759	0.5739	4.6027E-05	21.7
rs10805346	4	9920347	C	T	0.0117072	0.00202147	7.00E-09	-0.0216	0.1398	0.8772	3.49945E-05	16.5
rs10840270	11	9629553	G	C	0.014383	0.00212494	1.30E-11	-0.1616	0.1474	0.2731	4.38112E-05	20.7
rs10845387	12	11757743	A	G	-0.0141214	0.00209396	1.50E-11	-0.0467	0.151	0.757201	4.39783E-05	20.8
rs10905255	10	5870267	T	G	-0.0182493	0.00203099	2.60E-19	0.0565	0.1409	0.6883	8.33512E-05	39.4
rs11085072	19	4368142	T	C	-0.0131806	0.00236713	2.60E-08	-0.0936	0.1899	0.6222	2.37417E-05	11.2
rs11117354	16	88092092	C	T	0.0232506	0.00219601	3.40E-26	-0.073	0.1409	0.6045	0.000100368	47.4
rs111527438	17	29252703	C	T	0.0125	0.00211016	3.10E-09	0.2494	0.1497	0.0956005	3.38697E-05	16.0
rs111950327	16	48283993	C	G	0.0238271	0.00409406	5.90E-09	0.2811	0.3099	0.3644	8.54788E-06	4.0
rs112394943	3	197842892	C	T	-0.0198961	0.00281641	1.60E-12	-0.1443	0.1659	0.3844	2.88024E-05	13.6
rs113525195	14	23499321	A	C	-0.0124075	0.00224132	3.10E-08	-0.2011	0.1523	0.1867	2.67405E-05	12.6
rs11412296	15	50366116	T	TA	0.0332259	0.00234478	1.40E-45	-0.214	0.1628	0.1886	0.000155404	73.4
rs11557154	9	34107505	T	C	-0.0343719	0.00298538	1.10E-30	-0.1821	0.2062	0.3771	6.35161E-05	30.0
rs11579626	1	146741960	C	A	0.0265113	0.00357752	1.30E-13	0.1019	0.211	0.629001	1.80684E-05	8.5
rs11584821	1	114419489	T	C	-0.0306517	0.00263623	3.00E-31	0.3752	0.1809	0.0380996	8.31216E-05	39.3
rs116863223	18	709396	A	G	-0.0817874	0.00937157	2.60E-18	-0.0221	0.5668	0.9689	3.75021E-06	1.8
rs11699829	20	62157200	A	G	0.0641957	0.00602028	1.50E-26	0.2369	0.5656	0.6754	1.58839E-05	7.5
rs117034449	10	103961013	A	G	0.0374377	0.00667898	2.10E-08	0.1666	0.5484	0.7613	3.02912E-06	1.4

rs117407747	7	159117178	T	C	0.0450533	0.00611706	1.80E-13	1.0771	0.7349	0.1428	6.16036E-06	2.9
rs117512405	20	62574274	A	G	-0.0790134	0.00824611	9.50E-22	-0.0455	0.3177	0.8861	6.51496E-06	3.1
rs117630647	7	124779510	A	G	0.059565	0.00720413	1.40E-16	0.1296	0.5404	0.8104	6.04913E-06	2.9
rs11769630	7	50257703	A	T	-0.0256807	0.00389475	4.30E-11	0.4099	0.2289	0.0732706	1.23402E-05	5.8
rs11991877	8	56664524	A	T	-0.030138	0.00318686	3.20E-21	0.2201	0.1991	0.269	3.72903E-05	17.6
rs12369950	12	24762109	C	T	-0.0178308	0.00290205	8.00E-10	0.2868	0.1942	0.1398	1.93301E-05	9.1
rs12412214	10	101276256	A	G	-0.0245174	0.00222685	3.40E-28	-0.1949	0.1425	0.1715	0.000103458	48.9
rs12451892	17	2247982	C	T	-0.0116145	0.00207578	2.20E-08	0.1493	0.1411	0.2901	3.12584E-05	14.8
rs1291143	20	35525640	C	A	0.0493145	0.0027991	1.80E-69	0.0913	0.1674	0.5855	0.000168525	79.6
rs12925933	16	90141355	C	A	-0.0146622	0.00213796	7.00E-12	0.1328	0.1406	0.3448	4.45625E-05	21.0
rs12932179	16	9072085	G	A	-0.0136257	0.0020276	1.80E-11	0.1913	0.1434	0.1823	4.71001E-05	22.2
rs13062095	3	101267385	C	T	0.0138552	0.00214113	9.70E-11	0.072	0.1434	0.615601	3.90845E-05	18.5
rs13230646	7	23930316	C	T	-0.0173277	0.00232377	8.90E-14	-0.1606	0.1853	0.386	4.40351E-05	20.8
rs1332941	13	41695100	G	A	0.0256552	0.00273159	5.90E-21	-0.1832	0.1721	0.287	5.5037E-05	26.0
rs137901416	14	73418095	A	G	0.04572	0.00332355	4.70E-43	-0.1154	0.2856	0.6863	7.23397E-05	34.2
rs139669835	18	729871	T	C	-0.0612563	0.0105346	6.10E-09	-1.069	1.9	0.5737	1.32866E-06	0.6
rs139795227	1	92842367	C	A	0.0599379	0.00873247	6.70E-12	-0.851	0.4169	0.0412098	2.75869E-06	1.3
rs141214782	5	78954683	TTATC	T	-0.0246687	0.00335723	2.00E-13	-0.2315	0.2283	0.3104	2.08029E-05	9.8
rs142426306	20	62488152	T	C	-0.0504903	0.00539933	8.70E-21	0.1701	0.3643	0.6404	1.40677E-05	6.6
rs143190905	20	62291767	T	G	-0.0723995	0.00369421	1.60E-85	0.3469	0.2639	0.1886	0.00012029	56.8
rs144204502	17	76183233	T	C	-0.100574	0.00913369	3.40E-28	-0.1059	0.3668	0.7728	6.37053E-06	3.0
rs145114957	1	94322469	G	C	0.0272605	0.00498872	4.60E-08	0.6788	0.3508	0.0529798	5.16402E-06	2.4
rs150150565	18	708207	T	C	0.063762	0.00739877	6.80E-18	-0.4891	0.5268	0.3532	6.60453E-06	3.1
rs1611236	6	29748690	A	G	-0.0160135	0.00213359	6.10E-14	-0.1237	0.1711	0.4694	5.24992E-05	24.8
rs16978028	18	42070981	T	A	-0.029945	0.00285068	8.20E-26	0.0628	0.2534	0.8043	5.75215E-05	27.2
rs17445108	12	57082058	A	G	-0.0168922	0.00300983	2.00E-08	0.1105	0.2125	0.603	1.47873E-05	7.0
rs17677991	15	42032383	G	C	0.0222664	0.00210806	4.40E-26	-0.0975	0.1424	0.4935	0.000106363	50.2
rs17803849	2	210679608	T	C	0.0273203	0.00203482	4.20E-41	0.1018	0.1407	0.4693	0.000184024	86.9
rs182059586	16	14652220	C	T	-0.0571159	0.00680853	4.90E-17	1.2371	0.873	0.1564	7.29689E-06	3.4
rs185174247	5	138914024	A	G	0.0372806	0.00435145	1.10E-17	-0.3721	0.4099	0.364	1.64605E-05	7.8
rs188918174	2	54473646	T	C	0.0403062	0.00543604	1.20E-13	0.76	0.3282	0.0205698	8.10429E-06	3.8
rs1907702	12	88955469	A	G	0.0150247	0.00242651	5.90E-10	-0.0305	0.1727	0.8599	2.90418E-05	13.7
rs1957937	14	96181360	T	A	0.0209365	0.00273361	1.90E-14	-0.1984	0.1772	0.2629	3.34239E-05	15.8
rs1985369	7	159119220	G	A	-0.0311893	0.00300952	3.60E-25	-0.1194	0.2178	0.5836	5.20645E-05	24.6
rs2056726	7	99780283	A	G	-0.0228078	0.00243638	7.90E-21	-0.2174	0.1678	0.1951	6.25166E-05	29.5
rs2230590	3	49936102	C	T	-0.0158022	0.00200806	3.60E-15	-0.0871	0.1426	0.541499	6.55456E-05	31.0
rs2282764	4	2255063	G	A	-0.0224234	0.00289392	9.30E-15	-0.155	0.1984	0.4346	3.10536E-05	14.7
rs2538745	7	76310784	C	T	-0.012942	0.002056	3.10E-10	-0.0463	0.1394	0.7399	4.01839E-05	19.0
rs2555104	2	17841243	C	A	-0.0139717	0.00203498	6.60E-12	0.0459	0.1394	0.742099	4.90537E-05	23.2
rs28363070	5	1415068	A	G	0.0755557	0.00959987	3.50E-15	2.0338	1.8085	0.2608	3.46598E-06	1.6
rs28502153	22	17469049	A	C	-0.0215916	0.00206208	1.20E-25	-3.00E-04	0.1437	0.9986	0.000109182	51.6

rs28577594	12	123895906	C	G	0.0187657	0.00224024	5.40E-17	-0.1326	0.1542	0.3897	6.12176E-05	28.9
rs2967355	16	82200103	C	A	-0.0461595	0.00238972	4.00E-83	0.0795	0.1836	0.6652	0.000276204	130.5
rs2977608	1	768253	C	A	0.0129483	0.00233716	3.00E-08	0.1116	0.1522	0.4633	2.47654E-05	11.7
rs3093888	14	20812951	A	G	-0.028973	0.00452459	1.50E-10	0.0649	0.2415	0.788099	8.4544E-06	4.0
rs35446936	3	169486508	A	G	-0.0940025	0.00232918	1.00E-200	0.086	0.1564	0.5825	0.001271507	601.1
rs3767952	1	41231032	A	G	0.0134472	0.00238826	1.80E-08	0.0496	0.1667	0.765799	2.35418E-05	11.1
rs3785074	16	69406986	G	A	0.023863	0.00220455	2.60E-27	-0.1436	0.1722	0.4045	0.000102118	48.2
rs3891167	18	658423	G	A	-0.0425685	0.00239551	1.20E-70	-0.2111	0.1617	0.1919	0.000253071	119.5
rs41269079	1	45252015	A	T	0.0153617	0.0025499	1.70E-09	-0.1871	0.1717	0.2758	2.35628E-05	11.1
rs41304832	20	62375508	A	G	0.0611702	0.0093095	5.00E-11	0.0161	0.3642	0.9648	2.23561E-06	1.1
rs4498805	1	110910397	T	G	0.0150601	0.00200376	5.70E-14	-0.0045	0.1388	0.9742	5.92977E-05	28.0
rs4530278	19	33752994	T	G	0.0138793	0.0020567	1.50E-11	-0.0689	0.1426	0.6292	4.63655E-05	21.9
rs45604339	14	65543102	T	C	-0.020433	0.00211433	4.30E-22	0.3102	0.143	0.0300103	8.90747E-05	42.1
rs4616688	3	160042459	T	G	-0.0173476	0.00200198	4.50E-18	-0.2192	0.142	0.1228	7.93059E-05	37.4
rs4695407	4	48843372	G	A	0.0141511	0.00199925	1.50E-12	-0.2212	0.1394	0.1127	5.30405E-05	25.0
rs4724	17	7760397	A	G	-0.0547446	0.00312441	9.80E-69	-0.0048	0.2265	0.983	0.000133944	63.3
rs4731541	7	128678236	G	C	-0.0206119	0.00205962	1.40E-23	0.063	0.1442	0.6622	9.94367E-05	47.0
rs4743037	9	109639970	T	C	0.0147971	0.00238094	5.10E-10	-0.0445	0.1677	0.7906	2.90507E-05	13.7
rs5742915	15	74336633	C	T	0.0193377	0.00202886	1.60E-21	-0.2152	0.1414	0.1281	9.50704E-05	44.9
rs59409453	17	1666218	G	A	0.0202133	0.00230175	1.60E-18	-0.3655	0.1717	0.0332399	6.42926E-05	30.4
rs6007020	22	45790132	C	T	0.0144904	0.00209637	4.80E-12	-0.1104	0.1451	0.4467	4.70577E-05	22.2
rs6054257	20	66370	A	G	-0.0141684	0.00247729	1.10E-08	-4.00E-04	0.1757	0.9981	2.27012E-05	10.7
rs611646	11	108177097	A	T	-0.0368309	0.00203547	3.50E-73	-0.3013	0.1416	0.0333304	0.000335143	158.3
rs61405042	7	67200	T	C	-0.0501874	0.00602955	8.50E-17	0.4209	0.6541	0.5199	8.3431E-06	3.9
rs61748181	5	1294166	T	C	-0.059181	0.00595394	2.80E-23	0.2294	0.3051	0.452	1.17558E-05	5.6
rs6536702	4	164028105	A	G	0.0534148	0.00238875	9.40E-111	0.0078	0.18	0.9654	0.000369723	174.6
rs6584579	10	105645725	G	A	0.0114923	0.00204674	2.00E-08	-0.0035	0.1489	0.9812	3.20198E-05	15.1
rs6587577	1	151402045	G	A	-0.0182148	0.0026359	4.80E-12	0.099	0.1916	0.605399	2.90246E-05	13.7
rs6659669	1	185315067	T	C	-0.0117091	0.00205167	1.10E-08	-0.1826	0.1406	0.1943	3.29668E-05	15.6
rs6669563	1	32279629	A	G	0.0182358	0.00202476	2.10E-19	-0.0382	0.1399	0.7845	8.45649E-05	39.9
rs66731853	1	20916238	A	G	-0.0177791	0.00215421	1.50E-16	0.0238	0.1756	0.8921	6.24992E-05	29.5
rs6751209	2	43588302	C	T	-0.0140465	0.00248465	1.60E-08	0.2034	0.1808	0.2608	2.2001E-05	10.4
rs6776756	3	128215821	A	G	-0.0174439	0.00203747	1.10E-17	0.004	0.1401	0.9773	7.46646E-05	35.3
rs6790988	3	170263320	G	A	0.0145728	0.00228428	1.80E-10	-0.0068	0.1655	0.967	3.30101E-05	15.6
rs6881568	5	1670265	A	C	0.0169256	0.00207735	3.70E-16	0.1952	0.1565	0.2124	6.4987E-05	30.7
rs7099229	10	96134685	A	G	-0.0153288	0.00224403	8.40E-12	-0.1443	0.1826	0.4295	3.92539E-05	18.5
rs7164950	15	56775385	G	A	0.0129362	0.00204001	2.30E-10	-0.1871	0.1414	0.1856	4.10754E-05	19.4
rs7209057	17	65705530	A	G	0.011819	0.00202865	5.70E-09	0.0915	0.1399	0.5128	3.54073E-05	16.7
rs7221585	17	76195153	T	C	0.0143271	0.00247042	6.70E-09	-0.144	0.1536	0.3484	2.47643E-05	11.7
rs73581419	14	21941148	T	C	0.0229838	0.00324156	1.30E-12	0.0398	0.2528	0.875	2.02808E-05	9.6
rs73730598	5	77973	A	G	0.0273632	0.00439272	4.70E-10	-0.1521	0.2799	0.5868	8.51229E-06	4.0

rs76065543	16	74678063	T	C	0.0342843	0.00290708	4.20E-32	-0.0929	0.1802	0.606301	6.98776E-05	33.0
rs76219171	16	50188929	A	G	0.0359839	0.00431741	7.80E-17	0.4921	0.3753	0.1898	1.61885E-05	7.6
rs762679	8	48885436	A	T	0.0310104	0.00285024	1.40E-27	8.00E-04	0.1859	0.9967	6.16249E-05	29.1
rs76666449	12	120904895	C	T	0.0295125	0.00333186	8.20E-19	0.3605	0.2026	0.0752697	3.00755E-05	14.2
rs7705526	5	1285974	A	C	0.0776022	0.00216124	1.00E-200	-0.2475	0.15	0.09891	0.001201003	567.8
rs77231040	10	106280527	C	G	0.0989303	0.0134649	2.00E-13	-0.0062	0.4528	0.989	1.3054E-06	0.6
rs7772289	6	28674322	T	G	0.017549	0.00200003	1.70E-18	0.0643	0.1455	0.6584	8.15236E-05	38.5
rs77732866	2	58979879	A	G	0.0177942	0.00290595	9.20E-10	0.1289	0.1992	0.517399	1.88464E-05	8.9
rs7790856	7	124459852	T	C	-0.0437199	0.00220526	1.80E-87	-0.1465	0.1678	0.3826	0.000342183	161.6
rs78491606	3	72891547	C	A	-0.0756311	0.00741168	1.90E-24	0.2181	0.4334	0.6148	7.98016E-06	3.8
rs79977579	12	54694560	A	C	0.0281517	0.00343182	2.30E-16	0.0176	0.2391	0.9412	2.46322E-05	11.6
rs80116508	16	3650970	A	G	-0.0352672	0.00415151	2.00E-17	0.1061	0.2575	0.680299	1.7872E-05	8.4
rs80324517	6	204031	A	G	0.0396515	0.00466286	1.80E-17	0.5492	0.2887	0.0571097	1.40682E-05	6.6
rs8102497	19	57370055	A	G	-0.0149654	0.0020233	1.40E-13	-0.0275	0.1397	0.844	5.68558E-05	26.8
rs8105767	19	22215441	G	A	0.0328384	0.00220117	2.50E-50	-0.056	0.1483	0.7059	0.000195935	92.5
rs869785	3	24347800	C	T	-0.0147303	0.00212801	4.40E-12	0.1781	0.1473	0.2265	4.4702E-05	21.1
rs871134	4	7044380	T	C	-0.0182986	0.0020263	1.70E-19	-0.4269	0.1407	0.00241002	8.47106E-05	40.0
rs932002	1	226577306	T	C	-0.0402052	0.00279667	7.30E-47	0.2961	0.1637	0.0704904	0.00011213	53.0
rs9398196	6	109601554	G	A	-0.0143586	0.00201175	9.50E-13	0.0441	0.1444	0.759899	5.38574E-05	25.4
rs939916	11	202253	A	G	0.0241795	0.00216724	6.60E-29	0.0966	0.1572	0.539	0.000116579	55.1
rs9419958	10	105675946	C	T	-0.0810098	0.00293847	2.60E-167	0.1133	0.22	0.606599	0.000384375	181.6
rs9600019	13	73317585	T	C	0.0127134	0.00213096	2.40E-09	-0.069	0.144	0.631801	3.36154E-05	15.9
rs9940099	16	3613207	T	G	-0.033609	0.00411613	3.20E-16	0.0358	0.2575	0.8894	1.66006E-05	7.8
rs9955360	18	78008334	A	C	-0.0190311	0.00299791	2.20E-10	0.1999	0.1974	0.3112	1.93954E-05	9.2
**TL-PAD**												
rs1003322	22	51072289	A	C	0.0141734	0.00247546	1.00E-08	-0.0053	0.0289	0.8537	2.33356E-05	11.0
rs10112752	8	73958718	A	G	-0.0287522	0.00202518	9.50E-46	0.0137	0.0205	0.5046	0.000209304	98.8
rs1023767	8	95530969	A	G	-0.0183732	0.00234772	5.00E-15	0.0114	0.021	0.5884	4.69924E-05	22.2
rs10768683	11	5247791	G	C	0.0469922	0.00277015	1.50E-64	-0.0235	0.0252	0.3499	0.000162964	77.0
rs10773176	12	122944713	G	A	-0.0172009	0.00228534	5.20E-14	1.00E-04	0.0258	0.9957	4.6027E-05	21.7
rs10805346	4	9920347	C	T	0.0117072	0.00202147	7.00E-09	0.0021	0.0204	0.9174	3.49945E-05	16.5
rs10840270	11	9629553	G	C	0.014383	0.00212494	1.30E-11	0.0341	0.0216	0.1146	4.38112E-05	20.7
rs10845387	12	11757743	A	G	-0.0141214	0.00209396	1.50E-11	-0.0102	0.022	0.6422	4.39783E-05	20.8
rs10905255	10	5870267	T	G	-0.0182493	0.00203099	2.60E-19	0.007	0.0206	0.7334	8.33512E-05	39.4
rs11085072	19	4368142	T	C	-0.0131806	0.00236713	2.60E-08	-0.0131	0.0276	0.6343	2.37417E-05	11.2
rs11117354	16	88092092	C	T	0.0232506	0.00219601	3.40E-26	-0.0215	0.0206	0.2971	0.000100368	47.4
rs111527438	17	29252703	C	T	0.0125	0.00211016	3.10E-09	-0.0018	0.0219	0.9345	3.38697E-05	16.0
rs111950327	16	48283993	C	G	0.0238271	0.00409406	5.90E-09	-0.0082	0.0456	0.8576	8.54788E-06	4.0
rs112394943	3	197842892	C	T	-0.0198961	0.00281641	1.60E-12	-0.0525	0.0243	0.0305999	2.88024E-05	13.6
rs113525195	14	23499321	A	C	-0.0124075	0.00224132	3.10E-08	0.0244	0.0222	0.2708	2.67405E-05	12.6
rs11412296	15	50366116	T	TA	0.0332259	0.00234478	1.40E-45	0.0183	0.0239	0.4439	0.000155404	73.4

rs11557154	9	34107505	T	C	-0.0343719	0.00298538	1.10E-30	-0.0205	0.0299	0.4937	6.35161E-05	30.0
rs11579626	1	146741960	C	A	0.0265113	0.00357752	1.30E-13	-0.0165	0.0309	0.5924	1.80684E-05	8.5
rs11584821	1	114419489	T	C	-0.0306517	0.00263623	3.00E-31	-0.0175	0.0263	0.5051	8.31216E-05	39.3
rs116863223	18	709396	A	G	-0.0817874	0.00937157	2.60E-18	0.1924	0.0819	0.0187698	3.75021E-06	1.8
rs11699829	20	62157200	A	G	0.0641957	0.00602028	1.50E-26	-0.1323	0.0842	0.1161	1.58839E-05	7.5
rs117407747	7	159117178	T	C	0.0450533	0.00611706	1.80E-13	-0.0572	0.1081	0.596499	6.16036E-06	2.9
rs117512405	20	62574274	A	G	-0.0790134	0.00824611	9.50E-22	-0.0054	0.0453	0.9048	6.51496E-06	3.1
rs117630647	7	124779510	A	G	0.059565	0.00720413	1.40E-16	0.0184	0.0793	0.8169	6.04913E-06	2.9
rs11769630	7	50257703	A	T	-0.0256807	0.00389475	4.30E-11	0.041	0.0335	0.2213	1.23402E-05	5.8
rs11991877	8	56664524	A	T	-0.030138	0.00318686	3.20E-21	0.0362	0.0291	0.2133	3.72903E-05	17.6
rs12369950	12	24762109	C	T	-0.0178308	0.00290205	8.00E-10	0.012	0.0284	0.6736	1.93301E-05	9.1
rs12412214	10	101276256	A	G	-0.0245174	0.00222685	3.40E-28	0.0274	0.0208	0.1867	0.000103458	48.9
rs12451892	17	2247982	C	T	-0.0116145	0.00207578	2.20E-08	-0.0144	0.0206	0.4853	3.12584E-05	14.8
rs1291143	20	35525640	C	A	0.0493145	0.0027991	1.80E-69	0.0452	0.0245	0.0648007	0.000168525	79.6
rs12925933	16	90141355	C	A	-0.0146622	0.00213796	7.00E-12	0.0231	0.0204	0.2578	4.45625E-05	21.0
rs12932179	16	9072085	G	A	-0.0136257	0.0020276	1.80E-11	-0.0064	0.021	0.7596	4.71001E-05	22.2
rs13062095	3	101267385	C	T	0.0138552	0.00214113	9.70E-11	-0.004	0.021	0.8482	3.90845E-05	18.5
rs13230646	7	23930316	C	T	-0.0173277	0.00232377	8.90E-14	0.073	0.0271	0.00713001	4.40351E-05	20.8
rs1332941	13	41695100	G	A	0.0256552	0.00273159	5.90E-21	-0.0067	0.0251	0.7887	5.5037E-05	26.0
rs137901416	14	73418095	A	G	0.04572	0.00332355	4.70E-43	-0.0183	0.0412	0.6574	7.23397E-05	34.2
rs139669835	18	729871	T	C	-0.0612563	0.0105346	6.10E-09	-0.327	0.2931	0.2645	1.32866E-06	0.6
rs139795227	1	92842367	C	A	0.0599379	0.00873247	6.70E-12	-0.0849	0.0613	0.1657	2.75869E-06	1.3
rs141214782	5	78954683	TTATC	T	-0.0246687	0.00335723	2.00E-13	0.0126	0.0333	0.7042	2.08029E-05	9.8
rs142426306	20	62488152	T	C	-0.0504903	0.00539933	8.70E-21	-0.0672	0.0525	0.2011	1.40677E-05	6.6
rs143190905	20	62291767	T	G	-0.0723995	0.00369421	1.60E-85	4.00E-04	0.0386	0.9928	0.00012029	56.8
rs144204502	17	76183233	T	C	-0.100574	0.00913369	3.40E-28	0.0445	0.0536	0.4062	6.37053E-06	3.0
rs145114957	1	94322469	G	C	0.0272605	0.00498872	4.60E-08	0.0082	0.0521	0.8744	5.16402E-06	2.4
rs150150565	18	708207	T	C	0.063762	0.00739877	6.80E-18	-0.081	0.0757	0.2846	6.60453E-06	3.1
rs1611236	6	29748690	A	G	-0.0160135	0.00213359	6.10E-14	0.0067	0.0252	0.791099	5.24992E-05	24.8
rs16978028	18	42070981	T	A	-0.029945	0.00285068	8.20E-26	-0.0383	0.0371	0.3022	5.75215E-05	27.2
rs17445108	12	57082058	A	G	-0.0168922	0.00300983	2.00E-08	-0.0014	0.0311	0.9632	1.47873E-05	7.0
rs17677991	15	42032383	G	C	0.0222664	0.00210806	4.40E-26	0.0454	0.0208	0.0289201	0.000106363	50.2
rs182059586	16	14652220	C	T	-0.0571159	0.00680853	4.90E-17	0.0051	0.1309	0.9691	7.29689E-06	3.4
rs185174247	5	138914024	A	G	0.0372806	0.00435145	1.10E-17	0.0548	0.0599	0.3601	1.64605E-05	7.8
rs188918174	2	54473646	T	C	0.0403062	0.00543604	1.20E-13	0.0561	0.0487	0.2492	8.10429E-06	3.8
rs1907702	12	88955469	A	G	0.0150247	0.00242651	5.90E-10	-0.0441	0.0251	0.0789805	2.90418E-05	13.7
rs1957937	14	96181360	T	A	0.0209365	0.00273361	1.90E-14	0.0121	0.0261	0.6438	3.34239E-05	15.8
rs1985369	7	159119220	G	A	-0.0311893	0.00300952	3.60E-25	0.0096	0.0324	0.7662	5.20645E-05	24.6
rs2056726	7	99780283	A	G	-0.0228078	0.00243638	7.90E-21	-0.0134	0.0243	0.580899	6.25166E-05	29.5
rs2230590	3	49936102	C	T	-0.0158022	0.00200806	3.60E-15	-0.02	0.0208	0.3362	6.55456E-05	31.0
rs2282764	4	2255063	G	A	-0.0224234	0.00289392	9.30E-15	-0.0447	0.0295	0.1293	3.10536E-05	14.7

rs2538745	7	76310784	C	T	-0.012942	0.002056	3.10E-10	-0.0068	0.0204	0.736999	4.01839E-05	19.0
rs2555104	2	17841243	C	A	-0.0139717	0.00203498	6.60E-12	0.0026	0.0203	0.8978	4.90537E-05	23.2
rs28363070	5	1415068	A	G	0.0755557	0.00959987	3.50E-15	-0.1089	0.2431	0.6543	3.46598E-06	1.6
rs28502153	22	17469049	A	C	-0.0215916	0.00206208	1.20E-25	-0.0131	0.0209	0.531401	0.000109182	51.6
rs28577594	12	123895906	C	G	0.0187657	0.00224024	5.40E-17	0.0413	0.0225	0.0667206	6.12176E-05	28.9
rs2967355	16	82200103	C	A	-0.0461595	0.00238972	4.00E-83	0.0182	0.0269	0.498901	0.000276204	130.5
rs2977608	1	768253	C	A	0.0129483	0.00233716	3.00E-08	-0.0108	0.0221	0.6244	2.47654E-05	11.7
rs3093888	14	20812951	A	G	-0.028973	0.00452459	1.50E-10	0.0219	0.0356	0.5391	8.4544E-06	4.0
rs35446936	3	169486508	A	G	-0.0940025	0.00232918	1.00E-200	0.0134	0.0229	0.5598	0.001271507	601.1
rs3767952	1	41231032	A	G	0.0134472	0.00238826	1.80E-08	0.0308	0.0244	0.2068	2.35418E-05	11.1
rs3785074	16	69406986	G	A	0.023863	0.00220455	2.60E-27	0.0308	0.025	0.2181	0.000102118	48.2
rs3891167	18	658423	G	A	-0.0425685	0.00239551	1.20E-70	-0.0123	0.0237	0.6026	0.000253071	119.5
rs41269079	1	45252015	A	T	0.0153617	0.0025499	1.70E-09	0.0496	0.025	0.0472596	2.35628E-05	11.1
rs41304832	20	62375508	A	G	0.0611702	0.0093095	5.00E-11	0.1006	0.0524	0.05465	2.23561E-06	1.1
rs4498805	1	110910397	T	G	0.0150601	0.00200376	5.70E-14	0.0091	0.0203	0.6531	5.92977E-05	28.0
rs4530278	19	33752994	T	G	0.0138793	0.0020567	1.50E-11	0.0264	0.0208	0.2043	4.63655E-05	21.9
rs45604339	14	65543102	T	C	-0.020433	0.00211433	4.30E-22	0.0507	0.0208	0.0149799	8.90747E-05	42.1
rs4616688	3	160042459	T	G	-0.0173476	0.00200198	4.50E-18	0.0126	0.0207	0.5444	7.93059E-05	37.4
rs4695407	4	48843372	G	A	0.0141511	0.00199925	1.50E-12	0.0124	0.0203	0.5417	5.30405E-05	25.0
rs4724	17	7760397	A	G	-0.0547446	0.00312441	9.80E-69	0.0171	0.0332	0.6058	0.000133944	63.3
rs4731541	7	128678236	G	C	-0.0206119	0.00205962	1.40E-23	-0.0067	0.0211	0.7509	9.94367E-05	47.0
rs4743037	9	109639970	T	C	0.0147971	0.00238094	5.10E-10	-0.0046	0.0247	0.8523	2.90507E-05	13.7
rs5742915	15	74336633	C	T	0.0193377	0.00202886	1.60E-21	-0.0367	0.0206	0.0751502	9.50704E-05	44.9
rs59409453	17	1666218	G	A	0.0202133	0.00230175	1.60E-18	0.0343	0.0252	0.1728	6.42926E-05	30.4
rs6007020	22	45790132	C	T	0.0144904	0.00209637	4.80E-12	0.0016	0.0213	0.9395	4.70577E-05	22.2
rs6054257	20	66370	A	G	-0.0141684	0.00247729	1.10E-08	-0.0221	0.0255	0.3851	2.27012E-05	10.7
rs611646	11	108177097	A	T	-0.0368309	0.00203547	3.50E-73	-0.0011	0.0208	0.9594	0.000335143	158.3
rs61405042	7	67200	T	C	-0.0501874	0.00602955	8.50E-17	0.1175	0.0942	0.212	8.3431E-06	3.9
rs61748181	5	1294166	T	C	-0.059181	0.00595394	2.80E-23	-0.0513	0.0443	0.2477	1.17558E-05	5.6
rs6536702	4	164028105	A	G	0.0534148	0.00238875	9.40E-111	0.0336	0.026	0.1972	0.000369723	174.6
rs6584579	10	105645725	G	A	0.0114923	0.00204674	2.00E-08	0.0057	0.0216	0.7905	3.20198E-05	15.1
rs6587577	1	151402045	G	A	-0.0182148	0.0026359	4.80E-12	-0.0198	0.0278	0.4768	2.90246E-05	13.7
rs6659669	1	185315067	T	C	-0.0117091	0.00205167	1.10E-08	-0.0143	0.0205	0.4872	3.29668E-05	15.6
rs6669563	1	32279629	A	G	0.0182358	0.00202476	2.10E-19	-0.0175	0.0204	0.3905	8.45649E-05	39.9
rs66731853	1	20916238	A	G	-0.0177791	0.00215421	1.50E-16	0.03	0.0256	0.2409	6.24992E-05	29.5
rs6751209	2	43588302	C	T	-0.0140465	0.00248465	1.60E-08	0.0176	0.0263	0.5047	2.2001E-05	10.4
rs6776756	3	128215821	A	G	-0.0174439	0.00203747	1.10E-17	-0.0229	0.0205	0.2647	7.46646E-05	35.3
rs6790988	3	170263320	G	A	0.0145728	0.00228428	1.80E-10	-0.0093	0.0243	0.701799	3.30101E-05	15.6
rs6881568	5	1670265	A	C	0.0169256	0.00207735	3.70E-16	-1.00E-04	0.0229	0.9973	6.4987E-05	30.7
rs7099229	10	96134685	A	G	-0.0153288	0.00224403	8.40E-12	0.0264	0.0268	0.3239	3.92539E-05	18.5
rs7164950	15	56775385	G	A	0.0129362	0.00204001	2.30E-10	0.015	0.0206	0.4661	4.10754E-05	19.4

rs7209057	17	65705530	A	G	0.011819	0.00202865	5.70E-09	-0.0121	0.0205	0.5546	3.54073E-05	16.7
rs7221585	17	76195153	T	C	0.0143271	0.00247042	6.70E-09	0.0216	0.0226	0.34	2.47643E-05	11.7
rs73581419	14	21941148	T	C	0.0229838	0.00324156	1.30E-12	0.0991	0.0369	0.00722005	2.02808E-05	9.6
rs73730598	5	77973	A	G	0.0273632	0.00439272	4.70E-10	-0.0167	0.0406	0.6819	8.51229E-06	4.0
rs76065543	16	74678063	T	C	0.0342843	0.00290708	4.20E-32	0.0145	0.0264	0.582801	6.98776E-05	33.0
rs76219171	16	50188929	A	G	0.0359839	0.00431741	7.80E-17	0.067	0.0544	0.2175	1.61885E-05	7.6
rs762679	8	48885436	A	T	0.0310104	0.00285024	1.40E-27	-0.011	0.027	0.683299	6.16249E-05	29.1
rs76666449	12	120904895	C	T	0.0295125	0.00333186	8.20E-19	0.0066	0.0297	0.8249	3.00755E-05	14.2
rs7705526	5	1285974	A	C	0.0776022	0.00216124	1.00E-200	-0.0144	0.0219	0.5106	0.001201003	567.8
rs77231040	10	106280527	C	G	0.0989303	0.0134649	2.00E-13	-0.0275	0.0654	0.674	1.3054E-06	0.6
rs7772289	6	28674322	T	G	0.017549	0.00200003	1.70E-18	-0.0132	0.0213	0.533499	8.15236E-05	38.5
rs77732866	2	58979879	A	G	0.0177942	0.00290595	9.20E-10	-0.0025	0.0293	0.9326	1.88464E-05	8.9
rs7790856	7	124459852	T	C	-0.0437199	0.00220526	1.80E-87	0.0586	0.0244	0.0164502	0.000342183	161.6
rs78491606	3	72891547	C	A	-0.0756311	0.00741168	1.90E-24	0.1065	0.0616	0.0836893	7.98016E-06	3.8
rs79977579	12	54694560	A	C	0.0281517	0.00343182	2.30E-16	-0.0864	0.0351	0.0138998	2.46322E-05	11.6
rs80116508	16	3650970	A	G	-0.0352672	0.00415151	2.00E-17	-0.0473	0.0374	0.207	1.7872E-05	8.4
rs80324517	6	204031	A	G	0.0396515	0.00466286	1.80E-17	0.0082	0.0418	0.8435	1.40682E-05	6.6
rs8102497	19	57370055	A	G	-0.0149654	0.0020233	1.40E-13	-0.0223	0.0204	0.2739	5.68558E-05	26.8
rs8105767	19	22215441	G	A	0.0328384	0.00220117	2.50E-50	0.0017	0.0217	0.9358	0.000195935	92.5
rs869785	3	24347800	C	T	-0.0147303	0.00212801	4.40E-12	0.0175	0.0215	0.4154	4.4702E-05	21.1
rs871134	4	7044380	T	C	-0.0182986	0.0020263	1.70E-19	0.0017	0.0205	0.9336	8.47106E-05	40.0
rs932002	1	226577306	T	C	-0.0402052	0.00279667	7.30E-47	0.023	0.024	0.3386	0.00011213	53.0
rs9398196	6	109601554	G	A	-0.0143586	0.00201175	9.50E-13	-0.0248	0.021	0.2386	5.38574E-05	25.4
rs939916	11	202253	A	G	0.0241795	0.00216724	6.60E-29	0.0059	0.0229	0.7978	0.000116579	55.1
rs9419958	10	105675946	C	T	-0.0810098	0.00293847	2.60E-167	8.00E-04	0.0317	0.9794	0.000384375	181.6
rs9600019	13	73317585	T	C	0.0127134	0.00213096	2.40E-09	0.0062	0.021	0.769501	3.36154E-05	15.9
rs9940099	16	3613207	T	G	-0.033609	0.00411613	3.20E-16	-0.0465	0.0374	0.2143	1.66006E-05	7.8
rs9955360	18	78008334	A	C	-0.0190311	0.00299791	2.20E-10	-0.0083	0.029	0.773101	1.93954E-05	9.2
**TL-AS, excluding cerebral, coronary and PAD**												
rs1003322	22	51072289	A	C	0.0141734	0.00247546	1.00E-08	-0.0246	0.0273	0.3661	2.33356E-05	11.0
rs10112752	8	73958718	A	G	-0.0287522	0.00202518	9.50E-46	0.0246	0.0193	0.2031	0.000209304	98.8
rs1023767	8	95530969	A	G	-0.0183732	0.00234772	5.00E-15	0.0176	0.0198	0.3741	4.69924E-05	22.2
rs10768683	11	5247791	G	C	0.0469922	0.00277015	1.50E-64	-0.0128	0.0237	0.5886	0.000162964	77.0
rs10773176	12	122944713	G	A	-0.0172009	0.00228534	5.20E-14	0.0124	0.0242	0.6105	4.6027E-05	21.7
rs10805346	4	9920347	C	T	0.0117072	0.00202147	7.00E-09	0.0098	0.0192	0.6095	3.49945E-05	16.5
rs10840270	11	9629553	G	C	0.014383	0.00212494	1.30E-11	0.0342	0.0203	0.0925401	4.38112E-05	20.7
rs10845387	12	11757743	A	G	-0.0141214	0.00209396	1.50E-11	-0.0097	0.0207	0.6383	4.39783E-05	20.8
rs10905255	10	5870267	T	G	-0.0182493	0.00203099	2.60E-19	-4.00E-04	0.0194	0.9823	8.33512E-05	39.4
rs11085072	19	4368142	T	C	-0.0131806	0.00236713	2.60E-08	0.021	0.026	0.4212	2.37417E-05	11.2
rs11117354	16	88092092	C	T	0.0232506	0.00219601	3.40E-26	-0.0188	0.0194	0.3346	0.000100368	47.4
rs111527438	17	29252703	C	T	0.0125	0.00211016	3.10E-09	0.0024	0.0206	0.9053	3.38697E-05	16.0

rs111950327	16	48283993	C	G	0.0238271	0.00409406	5.90E-09	0.0027	0.043	0.9505	8.54788E-06	4.0
rs112394943	3	197842892	C	T	-0.0198961	0.00281641	1.60E-12	-0.0335	0.0229	0.1429	2.88024E-05	13.6
rs113525195	14	23499321	A	C	-0.0124075	0.00224132	3.10E-08	0.0149	0.0209	0.4775	2.67405E-05	12.6
rs11412296	15	50366116	T	TA	0.0332259	0.00234478	1.40E-45	0.0203	0.0226	0.3676	0.000155404	73.4
rs11557154	9	34107505	T	C	-0.0343719	0.00298538	1.10E-30	-0.0272	0.0283	0.3364	6.35161E-05	30.0
rs11579626	1	146741960	C	A	0.0265113	0.00357752	1.30E-13	-0.0114	0.0291	0.696101	1.80684E-05	8.5
rs11584821	1	114419489	T	C	-0.0306517	0.00263623	3.00E-31	-0.0066	0.0248	0.79	8.31216E-05	39.3
rs116863223	18	709396	A	G	-0.0817874	0.00937157	2.60E-18	0.1314	0.0775	0.0897594	3.75021E-06	1.8
rs11699829	20	62157200	A	G	0.0641957	0.00602028	1.50E-26	-0.1228	0.079	0.1201	1.58839E-05	7.5
rs117034449	10	103961013	A	G	0.0374377	0.00667898	2.10E-08	0.0124	0.0771	0.8718	3.02912E-06	1.4
rs117407747	7	159117178	T	C	0.0450533	0.00611706	1.80E-13	-0.0346	0.1022	0.735099	6.16036E-06	2.9
rs117512405	20	62574274	A	G	-0.0790134	0.00824611	9.50E-22	-0.0069	0.0428	0.8714	6.51496E-06	3.1
rs117630647	7	124779510	A	G	0.059565	0.00720413	1.40E-16	0.0679	0.0754	0.3674	6.04913E-06	2.9
rs11769630	7	50257703	A	T	-0.0256807	0.00389475	4.30E-11	0.0302	0.0315	0.3382	1.23402E-05	5.8
rs11991877	8	56664524	A	T	-0.030138	0.00318686	3.20E-21	0.043	0.0273	0.1156	3.72903E-05	17.6
rs12369950	12	24762109	C	T	-0.0178308	0.00290205	8.00E-10	-0.0119	0.0267	0.6574	1.93301E-05	9.1
rs12412214	10	101276256	A	G	-0.0245174	0.00222685	3.40E-28	0.0177	0.0196	0.3663	0.000103458	48.9
rs12451892	17	2247982	C	T	-0.0116145	0.00207578	2.20E-08	-0.0194	0.0195	0.3175	3.12584E-05	14.8
rs1291143	20	35525640	C	A	0.0493145	0.0027991	1.80E-69	0.0425	0.023	0.0650894	0.000168525	79.6
rs12925933	16	90141355	C	A	-0.0146622	0.00213796	7.00E-12	0.03	0.0193	0.1202	4.45625E-05	21.0
rs12932179	16	9072085	G	A	-0.0136257	0.0020276	1.80E-11	-0.0029	0.0198	0.8845	4.71001E-05	22.2
rs13062095	3	101267385	C	T	0.0138552	0.00214113	9.70E-11	0.0136	0.0198	0.4932	3.90845E-05	18.5
rs13230646	7	23930316	C	T	-0.0173277	0.00232377	8.90E-14	0.052	0.0255	0.0412696	4.40351E-05	20.8
rs1332941	13	41695100	G	A	0.0256552	0.00273159	5.90E-21	0.0019	0.0237	0.9379	5.5037E-05	26.0
rs137901416	14	73418095	A	G	0.04572	0.00332355	4.70E-43	0.0248	0.0391	0.525801	7.23397E-05	34.2
rs139669835	18	729871	T	C	-0.0612563	0.0105346	6.10E-09	-0.5532	0.2665	0.0378896	1.32866E-06	0.6
rs139795227	1	92842367	C	A	0.0599379	0.00873247	6.70E-12	-0.0481	0.058	0.4066	2.75869E-06	1.3
rs141214782	5	78954683	TTATC	T	-0.0246687	0.00335723	2.00E-13	0.0253	0.0312	0.4177	2.08029E-05	9.8
rs142426306	20	62488152	T	C	-0.0504903	0.00539933	8.70E-21	-0.0058	0.0498	0.9076	1.40677E-05	6.6
rs143190905	20	62291767	T	G	-0.0723995	0.00369421	1.60E-85	0.0054	0.0362	0.881	0.00012029	56.8
rs144204502	17	76183233	T	C	-0.100574	0.00913369	3.40E-28	0.0466	0.0506	0.3577	6.37053E-06	3.0
rs145114957	1	94322469	G	C	0.0272605	0.00498872	4.60E-08	0.0073	0.0489	0.8816	5.16402E-06	2.4
rs150150565	18	708207	T	C	0.063762	0.00739877	6.80E-18	-0.0595	0.0716	0.4059	6.60453E-06	3.1
rs1611236	6	29748690	A	G	-0.0160135	0.00213359	6.10E-14	0.0161	0.0238	0.4996	5.24992E-05	24.8
rs16978028	18	42070981	T	A	-0.029945	0.00285068	8.20E-26	-0.0396	0.0349	0.2568	5.75215E-05	27.2
rs17445108	12	57082058	A	G	-0.0168922	0.00300983	2.00E-08	-0.0079	0.0292	0.786901	1.47873E-05	7.0
rs17677991	15	42032383	G	C	0.0222664	0.00210806	4.40E-26	0.0358	0.0196	0.0677002	0.000106363	50.2
rs17803849	2	210679608	T	C	0.0273203	0.00203482	4.20E-41	-0.0164	0.0194	0.3975	0.000184024	86.9
rs182059586	16	14652220	C	T	-0.0571159	0.00680853	4.90E-17	-0.0525	0.1214	0.6652	7.29689E-06	3.4
rs185174247	5	138914024	A	G	0.0372806	0.00435145	1.10E-17	0.0189	0.0573	0.7413	1.64605E-05	7.8
rs188918174	2	54473646	T	C	0.0403062	0.00543604	1.20E-13	0.0269	0.0453	0.552699	8.10429E-06	3.8

rs1907702	12	88955469	A	G	0.0150247	0.00242651	5.90E-10	-0.0408	0.0238	0.0866902	2.90418E-05	13.7
rs1957937	14	96181360	T	A	0.0209365	0.00273361	1.90E-14	0.011	0.0245	0.6529	3.34239E-05	15.8
rs1985369	7	159119220	G	A	-0.0311893	0.00300952	3.60E-25	0.03	0.0303	0.3221	5.20645E-05	24.6
rs2056726	7	99780283	A	G	-0.0228078	0.00243638	7.90E-21	-0.0172	0.023	0.4552	6.25166E-05	29.5
rs2230590	3	49936102	C	T	-0.0158022	0.00200806	3.60E-15	-0.0043	0.0196	0.8273	6.55456E-05	31.0
rs2282764	4	2255063	G	A	-0.0224234	0.00289392	9.30E-15	-0.0673	0.0275	0.01457	3.10536E-05	14.7
rs2538745	7	76310784	C	T	-0.012942	0.002056	3.10E-10	-0.0127	0.0192	0.5091	4.01839E-05	19.0
rs2555104	2	17841243	C	A	-0.0139717	0.00203498	6.60E-12	0	0.0192	0.9997	4.90537E-05	23.2
rs28363070	5	1415068	A	G	0.0755557	0.00959987	3.50E-15	-0.1785	0.2321	0.4419	3.46598E-06	1.6
rs28502153	22	17469049	A	C	-0.0215916	0.00206208	1.20E-25	-0.0092	0.0198	0.6401	0.000109182	51.6
rs28577594	12	123895906	C	G	0.0187657	0.00224024	5.40E-17	0.0299	0.0212	0.1582	6.12176E-05	28.9
rs2967355	16	82200103	C	A	-0.0461595	0.00238972	4.00E-83	0.0218	0.0253	0.3898	0.000276204	130.5
rs2977608	1	768253	C	A	0.0129483	0.00233716	3.00E-08	-0.0309	0.0209	0.1394	2.47654E-05	11.7
rs3093888	14	20812951	A	G	-0.028973	0.00452459	1.50E-10	0.0449	0.0334	0.1794	8.4544E-06	4.0
rs35446936	3	169486508	A	G	-0.0940025	0.00232918	1.00E-200	0.017	0.0216	0.4297	0.001271507	601.1
rs3767952	1	41231032	A	G	0.0134472	0.00238826	1.80E-08	0.016	0.023	0.4862	2.35418E-05	11.1
rs3785074	16	69406986	G	A	0.023863	0.00220455	2.60E-27	0.0669	0.0237	0.00470695	0.000102118	48.2
rs3891167	18	658423	G	A	-0.0425685	0.00239551	1.20E-70	-0.0202	0.0223	0.3662	0.000253071	119.5
rs41269079	1	45252015	A	T	0.0153617	0.0025499	1.70E-09	0.0448	0.0236	0.0572704	2.35628E-05	11.1
rs41304832	20	62375508	A	G	0.0611702	0.0093095	5.00E-11	0.1615	0.0497	0.001148	2.23561E-06	1.1
rs4498805	1	110910397	T	G	0.0150601	0.00200376	5.70E-14	0.0026	0.0191	0.8937	5.92977E-05	28.0
rs4530278	19	33752994	T	G	0.0138793	0.0020567	1.50E-11	0.0118	0.0196	0.5477	4.63655E-05	21.9
rs45604339	14	65543102	T	C	-0.020433	0.00211433	4.30E-22	0.0443	0.0196	0.0241102	8.90747E-05	42.1
rs4616688	3	160042459	T	G	-0.0173476	0.00200198	4.50E-18	0.0108	0.0196	0.581599	7.93059E-05	37.4
rs4695407	4	48843372	G	A	0.0141511	0.00199925	1.50E-12	0.0075	0.0192	0.6938	5.30405E-05	25.0
rs4724	17	7760397	A	G	-0.0547446	0.00312441	9.80E-69	0.0196	0.0312	0.5298	0.000133944	63.3
rs4731541	7	128678236	G	C	-0.0206119	0.00205962	1.40E-23	0.0035	0.0199	0.8603	9.94367E-05	47.0
rs4743037	9	109639970	T	C	0.0147971	0.00238094	5.10E-10	-0.0014	0.0232	0.9512	2.90507E-05	13.7
rs5742915	15	74336633	C	T	0.0193377	0.00202886	1.60E-21	-0.0412	0.0194	0.0342997	9.50704E-05	44.9
rs59409453	17	1666218	G	A	0.0202133	0.00230175	1.60E-18	0.048	0.0237	0.0426197	6.42926E-05	30.4
rs6007020	22	45790132	C	T	0.0144904	0.00209637	4.80E-12	0.0093	0.02	0.643899	4.70577E-05	22.2
rs6054257	20	66370	A	G	-0.0141684	0.00247729	1.10E-08	0.0017	0.024	0.944	2.27012E-05	10.7
rs611646	11	108177097	A	T	-0.0368309	0.00203547	3.50E-73	-0.0172	0.0195	0.3781	0.000335143	158.3
rs61405042	7	67200	T	C	-0.0501874	0.00602955	8.50E-17	0.0335	0.0873	0.701	8.3431E-06	3.9
rs61748181	5	1294166	T	C	-0.059181	0.00595394	2.80E-23	-0.0211	0.042	0.615	1.17558E-05	5.6
rs6536702	4	164028105	A	G	0.0534148	0.00238875	9.40E-111	0.0201	0.0246	0.4141	0.000369723	174.6
rs6584579	10	105645725	G	A	0.0114923	0.00204674	2.00E-08	0.002	0.0204	0.923	3.20198E-05	15.1
rs6587577	1	151402045	G	A	-0.0182148	0.0026359	4.80E-12	-0.0169	0.0263	0.5203	2.90246E-05	13.7
rs6659669	1	185315067	T	C	-0.0117091	0.00205167	1.10E-08	-0.0195	0.0193	0.3139	3.29668E-05	15.6
rs6669563	1	32279629	A	G	0.0182358	0.00202476	2.10E-19	-0.0141	0.0193	0.4636	8.45649E-05	39.9
rs66731853	1	20916238	A	G	-0.0177791	0.00215421	1.50E-16	0.017	0.0241	0.480799	6.24992E-05	29.5

rs6751209	2	43588302	C	T	-0.0140465	0.00248465	1.60E-08	0.0109	0.0248	0.6595	2.2001E-05	10.4
rs6776756	3	128215821	A	G	-0.0174439	0.00203747	1.10E-17	-0.035	0.0193	0.0707099	7.46646E-05	35.3
rs6790988	3	170263320	G	A	0.0145728	0.00228428	1.80E-10	0.0058	0.0228	0.7987	3.30101E-05	15.6
rs6881568	5	1670265	A	C	0.0169256	0.00207735	3.70E-16	-0.004	0.0215	0.8542	6.4987E-05	30.7
rs7099229	10	96134685	A	G	-0.0153288	0.00224403	8.40E-12	-0.0015	0.0252	0.9535	3.92539E-05	18.5
rs7164950	15	56775385	G	A	0.0129362	0.00204001	2.30E-10	0.0205	0.0194	0.2927	4.10754E-05	19.4
rs7209057	17	65705530	A	G	0.011819	0.00202865	5.70E-09	-0.0186	0.0193	0.3358	3.54073E-05	16.7
rs7221585	17	76195153	T	C	0.0143271	0.00247042	6.70E-09	0.0018	0.0213	0.9343	2.47643E-05	11.7
rs73581419	14	21941148	T	C	0.0229838	0.00324156	1.30E-12	0.0834	0.0349	0.0167201	2.02808E-05	9.6
rs73730598	5	77973	A	G	0.0273632	0.00439272	4.70E-10	0.0015	0.0383	0.9695	8.51229E-06	4.0
rs76065543	16	74678063	T	C	0.0342843	0.00290708	4.20E-32	-0.0033	0.0249	0.8953	6.98776E-05	33.0
rs76219171	16	50188929	A	G	0.0359839	0.00431741	7.80E-17	0.0366	0.0511	0.4743	1.61885E-05	7.6
rs762679	8	48885436	A	T	0.0310104	0.00285024	1.40E-27	-0.0279	0.0254	0.2724	6.16249E-05	29.1
rs76666449	12	120904895	C	T	0.0295125	0.00333186	8.20E-19	-0.0061	0.028	0.8279	3.00755E-05	14.2
rs7705526	5	1285974	A	C	0.0776022	0.00216124	1.00E-200	-0.0321	0.0206	0.1201	0.001201003	567.8
rs77231040	10	106280527	C	G	0.0989303	0.0134649	2.00E-13	-0.0304	0.0617	0.6226	1.3054E-06	0.6
rs7772289	6	28674322	T	G	0.017549	0.00200003	1.70E-18	0.0034	0.0201	0.8641	8.15236E-05	38.5
rs77732866	2	58979879	A	G	0.0177942	0.00290595	9.20E-10	-0.0202	0.0276	0.462701	1.88464E-05	8.9
rs7790856	7	124459852	T	C	-0.0437199	0.00220526	1.80E-87	0.0427	0.0231	0.0641298	0.000342183	161.6
rs78491606	3	72891547	C	A	-0.0756311	0.00741168	1.90E-24	0.0179	0.0579	0.758	7.98016E-06	3.8
rs79977579	12	54694560	A	C	0.0281517	0.00343182	2.30E-16	-0.0554	0.0331	0.0946804	2.46322E-05	11.6
rs80116508	16	3650970	A	G	-0.0352672	0.00415151	2.00E-17	-0.0536	0.0352	0.128	1.7872E-05	8.4
rs80324517	6	204031	A	G	0.0396515	0.00466286	1.80E-17	-0.0121	0.0395	0.7587	1.40682E-05	6.6
rs8102497	19	57370055	A	G	-0.0149654	0.0020233	1.40E-13	-0.0151	0.0192	0.4325	5.68558E-05	26.8
rs8105767	19	22215441	G	A	0.0328384	0.00220117	2.50E-50	0.0125	0.0205	0.5429	0.000195935	92.5
rs869785	3	24347800	C	T	-0.0147303	0.00212801	4.40E-12	0.0276	0.0202	0.1721	4.4702E-05	21.1
rs871134	4	7044380	T	C	-0.0182986	0.0020263	1.70E-19	0.0068	0.0193	0.724399	8.47106E-05	40.0
rs932002	1	226577306	T	C	-0.0402052	0.00279667	7.30E-47	0.0084	0.0226	0.7106	0.00011213	53.0
rs9398196	6	109601554	G	A	-0.0143586	0.00201175	9.50E-13	-0.0099	0.0199	0.618899	5.38574E-05	25.4
rs939916	11	202253	A	G	0.0241795	0.00216724	6.60E-29	0.0103	0.0217	0.6364	0.000116579	55.1
rs9419958	10	105675946	C	T	-0.0810098	0.00293847	2.60E-167	-0.0317	0.03	0.2915	0.000384375	181.6
rs9600019	13	73317585	T	C	0.0127134	0.00213096	2.40E-09	0.0047	0.0198	0.812	3.36154E-05	15.9
rs9940099	16	3613207	T	G	-0.033609	0.00411613	3.20E-16	-0.0526	0.0352	0.135	1.66006E-05	7.8
rs9955360	18	78008334	A	C	-0.0190311	0.00299791	2.20E-10	-0.0035	0.0272	0.8972	1.93954E-05	9.2
