# Supplementary material for: Assessment of the relationship between telomere length and atherosclerosis: A Mendelian randomization study
Source: Medicine (Baltimore). 2023 Nov 17;102(46):e35875. doi: 10.1097/MD.0000000000035875 (PMC10659599; doi:10.1097/MD.0000000000035875)
Supplement: Supplementary file 2 [file medi-102-e35875-s002.pdf]

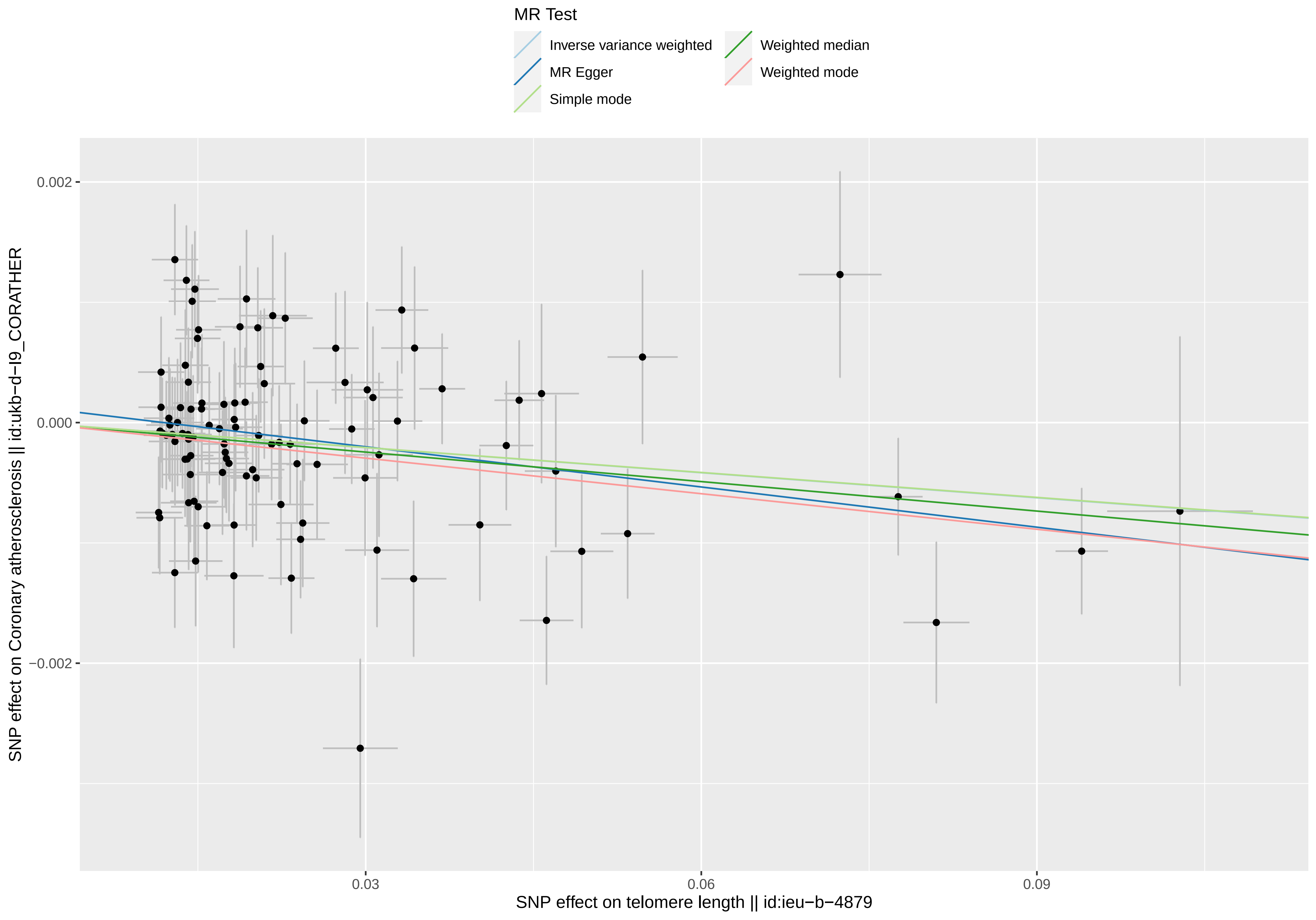

MR Test
Inverse variance weighted
MR Egger
Simple mode
Weighted median
Weighted mode
0.002
0.000
−0.002
0.03
0.06
0.09
SNP effect on telomere length || id:ieu−b−4879
SNP effect on Coronary atherosclerosis || id:ukb−d−I9_CORATHER

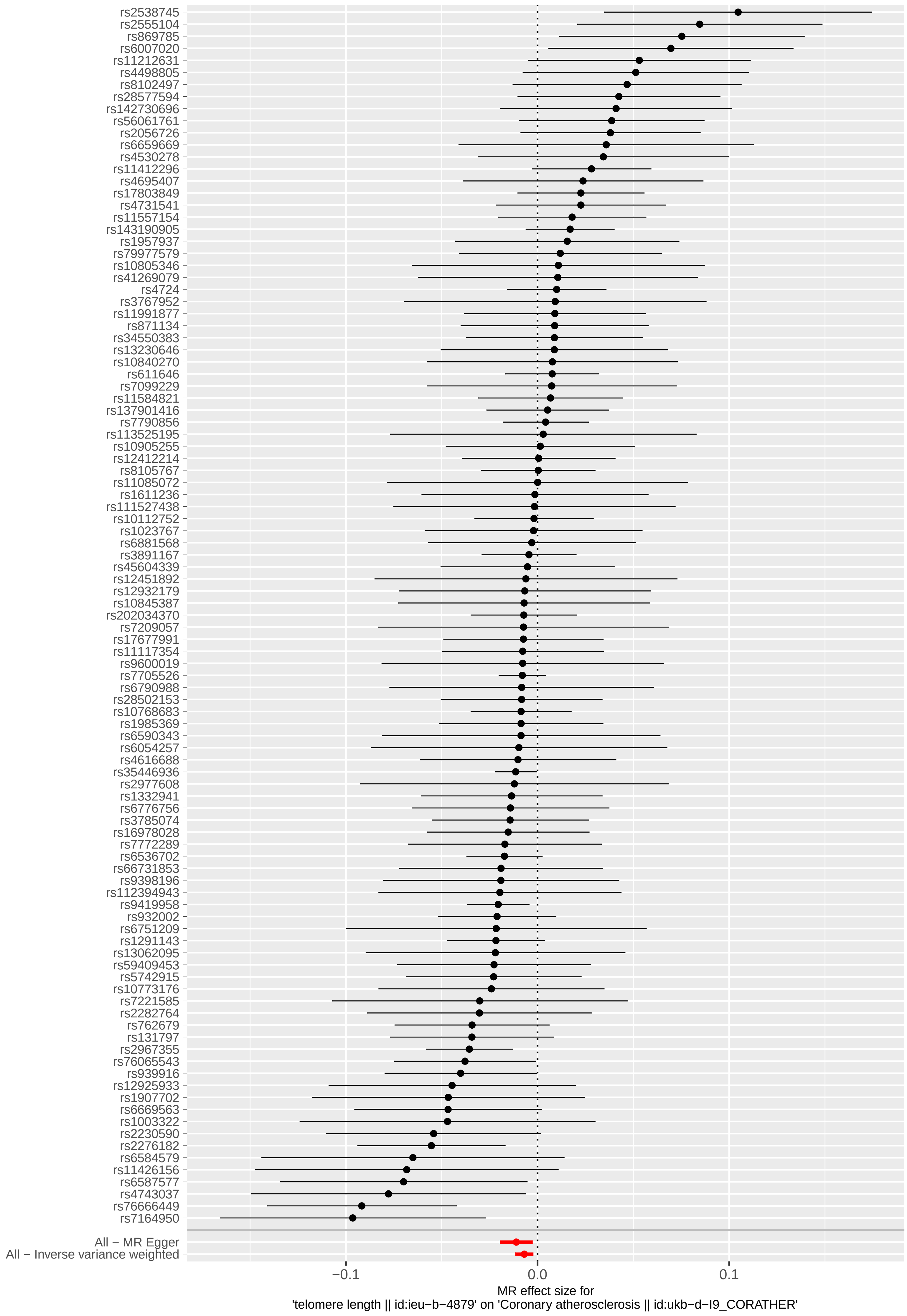
rs2538745
rs2555104
rs869785
rs6007020
rs11212631
rs4498805
rs8102497
rs28577594
rs142730696
rs56061761
rs2056726
rs6659669
rs4530278
rs11412296
rs4695407
rs17803849
rs4731541
rs11557154
rs143190905
rs1957937
rs79977579
rs10805346
rs41269079
rs4724
rs3767952
rs11991877
rs871134
rs34550383
rs13230646
rs10840270
rs611646
rs7099229
rs11584821
rs137901416
rs7790856
rs113525195
rs10905255
rs12412214
rs8105767
rs11085072
rs1611236
rs111527438
rs10112752
rs1023767
rs6881568
rs3891167
rs45604339
rs12451892
rs12932179
rs10845387
rs202034370
rs7209057
rs17677991
rs11117354
rs9600019
rs7705526
rs6790988
rs28502153
rs10768683
rs1985369
rs6590343
rs6054257
rs4616688
rs35446936
rs2977608
rs1332941
rs6776756
rs3785074
rs16978028
rs7772289
rs6536702
rs66731853
rs9398196
rs112394943
rs9419958
rs932002
rs6751209
rs1291143
rs13062095
rs59409453
rs5742915
rs10773176
rs7221585
rs2282764
rs762679
rs131797
rs2967355
rs76065543
rs939916
rs12925933
rs1907702
rs6669563
rs1003322
rs2230590
rs2276182
rs6584579
rs11426156
rs6587577
rs4743037
rs76666449
rs7164950
All − MR Egger
All − Inverse variance weighted
−0.1
0.0
0.1
MR effect size for
'telomere length || id:ieu−b−4879' on 'Coronary atherosclerosis || id:ukb−d−I9_CORATHER'

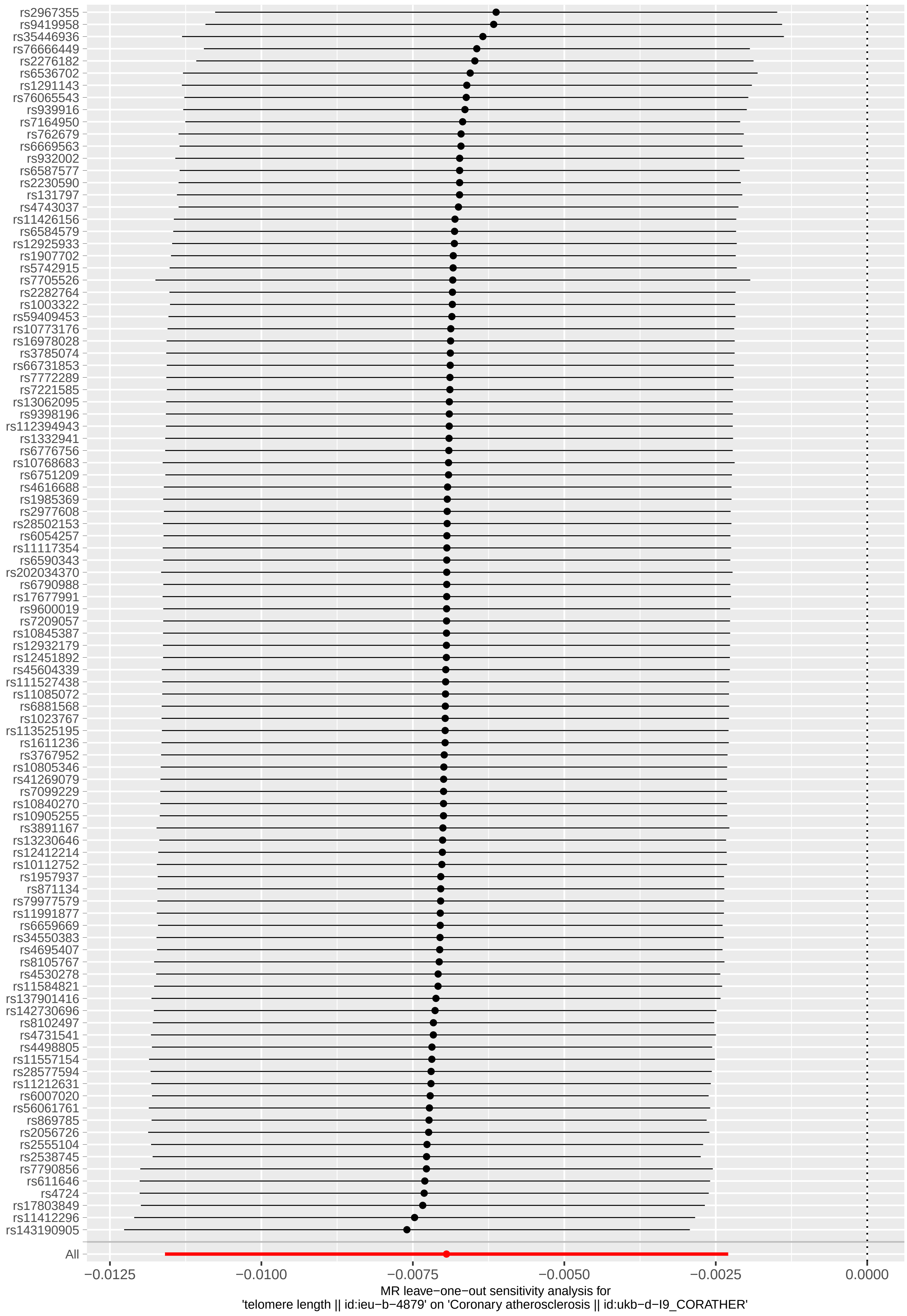
rs2967355
rs9419958
rs35446936
rs76666449
rs2276182
rs6536702
rs1291143
rs76065543
rs939916
rs7164950
rs762679
rs6669563
rs932002
rs6587577
rs2230590
rs131797
rs4743037
rs11426156
rs6584579
rs12925933
rs1907702
rs5742915
rs7705526
rs2282764
rs1003322
rs59409453
rs10773176
rs16978028
rs3785074
rs66731853
rs7772289
rs7221585
rs13062095
rs9398196
rs112394943
rs1332941
rs6776756
rs10768683
rs6751209
rs4616688
rs1985369
rs2977608
rs28502153
rs6054257
rs11117354
rs6590343
rs202034370
rs6790988
rs17677991
rs9600019
rs7209057
rs10845387
rs12932179
rs12451892
rs45604339
rs111527438
rs11085072
rs6881568
rs1023767
rs113525195
rs1611236
rs3767952
rs10805346
rs41269079
rs7099229
rs10840270
rs10905255
rs3891167
rs13230646
rs12412214
rs10112752
rs1957937
rs871134
rs79977579
rs11991877
rs6659669
rs34550383
rs4695407
rs8105767
rs4530278
rs11584821
rs137901416
rs142730696
rs8102497
rs4731541
rs4498805
rs11557154
rs28577594
rs11212631
rs6007020
rs56061761
rs869785
rs2056726
rs2555104
rs2538745
rs7790856
rs611646
rs4724
rs17803849
rs11412296
rs143190905
All
−0.0125
−0.0100
−0.0075
−0.0050
−0.0025
0.0000
MR leave−one−out sensitivity analysis for
'telomere length || id:ieu−b−4879' on 'Coronary atherosclerosis || id:ukb−d−I9_CORATHER'

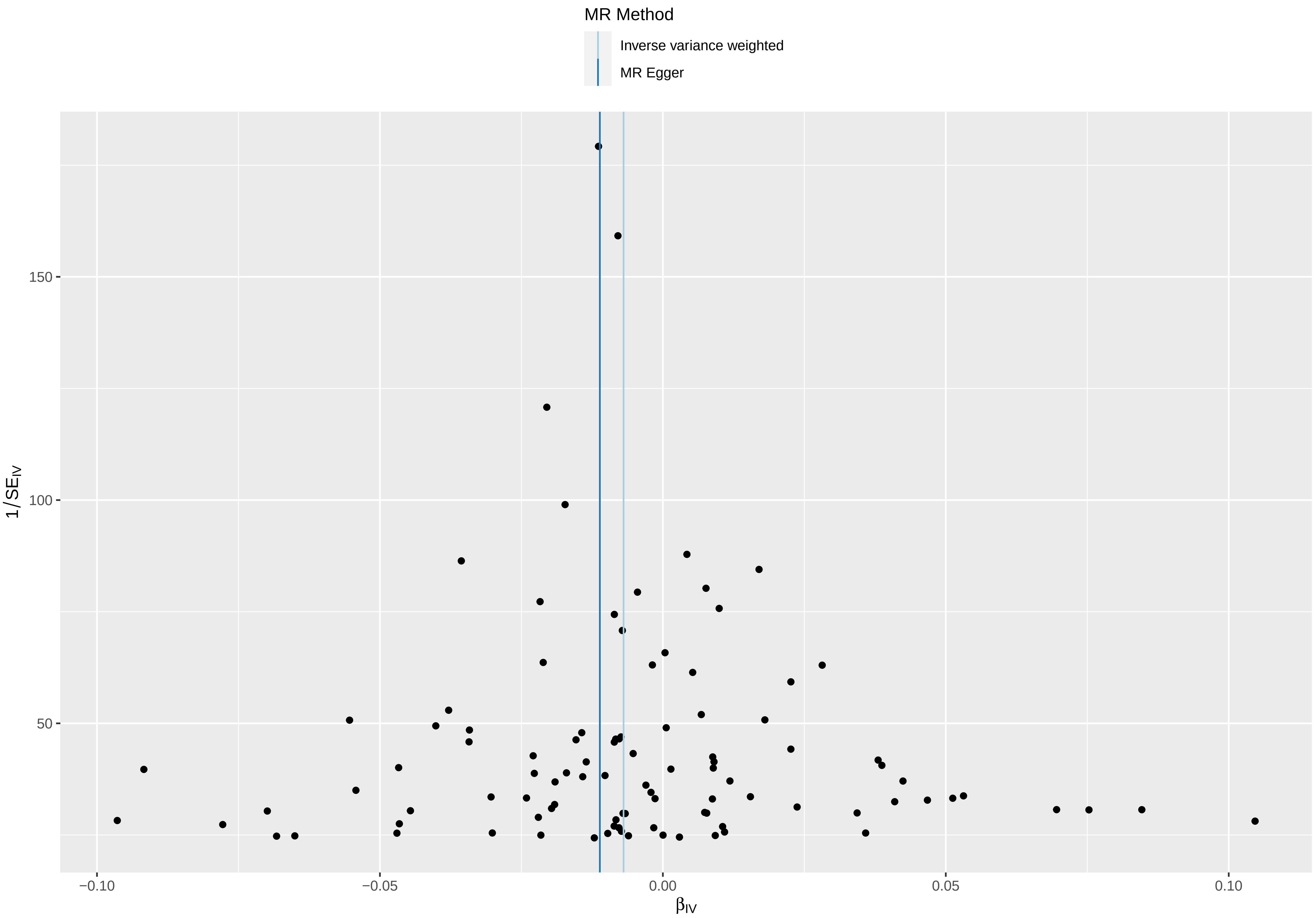

MR Method
Inverse variance weighted
MR Egger
1/SE$_{IV}$
150
100
50
−0.10
−0.05
0.00
0.05
0.10
β$_{IV}$

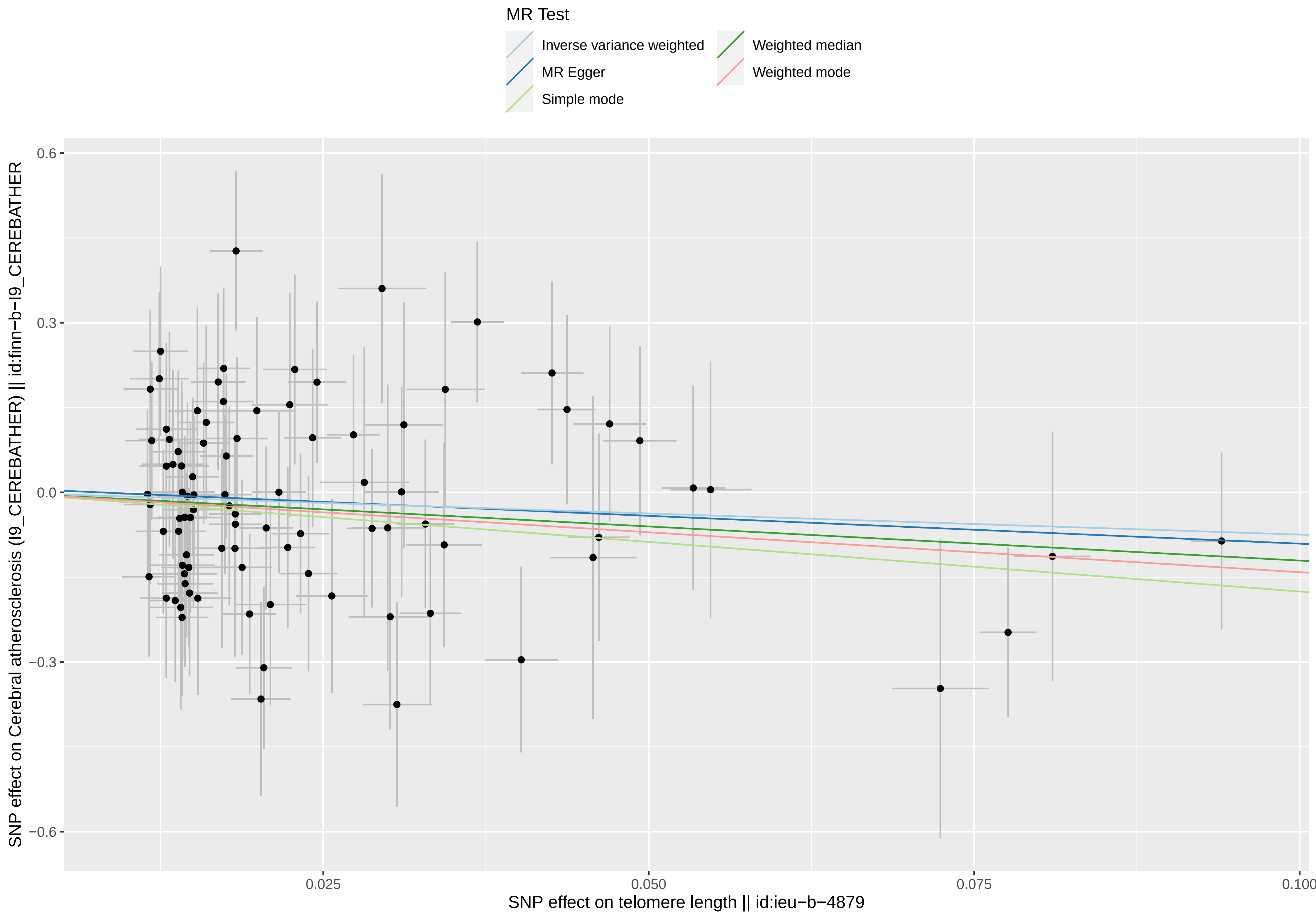
MR Test
Inverse variance weighted
MR Egger
Simple mode
Weighted median
Weighted mode
0.6
0.3
0.0
−0.3
−0.6
SNP effect on Cerebral atherosclerosis (I9_CEREBATHER) || id:finn−b−I9_CEREBATHER
0.025
0.050
0.075
0.100
SNP effect on telomere length || id:ieu−b−4879

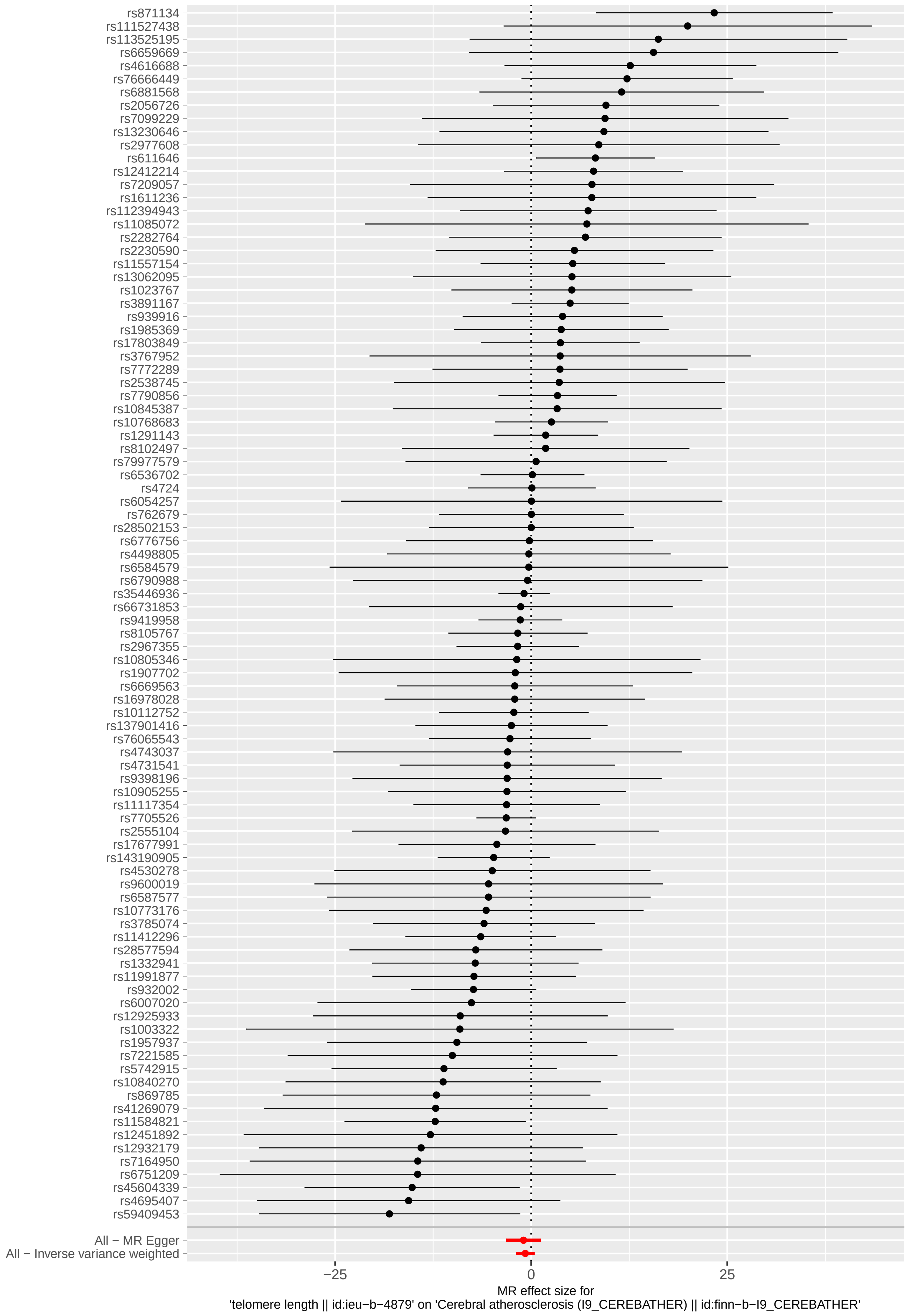

rs871134
rs111527438
rs113525195
rs6659669
rs4616688
rs76666449
rs6881568
rs2056726
rs7099229
rs13230646
rs2977608
rs611646
rs12412214
rs7209057
rs1611236
rs112394943
rs11085072
rs2282764
rs2230590
rs11557154
rs13062095
rs1023767
rs3891167
rs939916
rs1985369
rs17803849
rs3767952
rs7772289
rs2538745
rs7790856
rs10845387
rs10768683
rs1291143
rs8102497
rs79977579
rs6536702
rs4724
rs6054257
rs762679
rs28502153
rs6776756
rs4498805
rs6584579
rs6790988
rs35446936
rs66731853
rs9419958
rs8105767
rs2967355
rs10805346
rs1907702
rs6669563
rs16978028
rs10112752
rs137901416
rs76065543
rs4743037
rs4731541
rs9398196
rs10905255
rs11117354
rs7705526
rs2555104
rs17677991
rs143190905
rs4530278
rs9600019
rs6587577
rs10773176
rs3785074
rs11412296
rs28577594
rs1332941
rs11991877
rs932002
rs6007020
rs12925933
rs1003322
rs1957937
rs7221585
rs5742915
rs10840270
rs869785
rs41269079
rs11584821
rs12451892
rs12932179
rs7164950
rs6751209
rs45604339
rs4695407
rs59409453
All − MR Egger
All − Inverse variance weighted
−25
0
25
MR effect size for
'telomere length || id:ieu−b−4879' on 'Cerebral atherosclerosis (I9_CEREBATHER) || id:finn−b−I9_CEREBATHER'

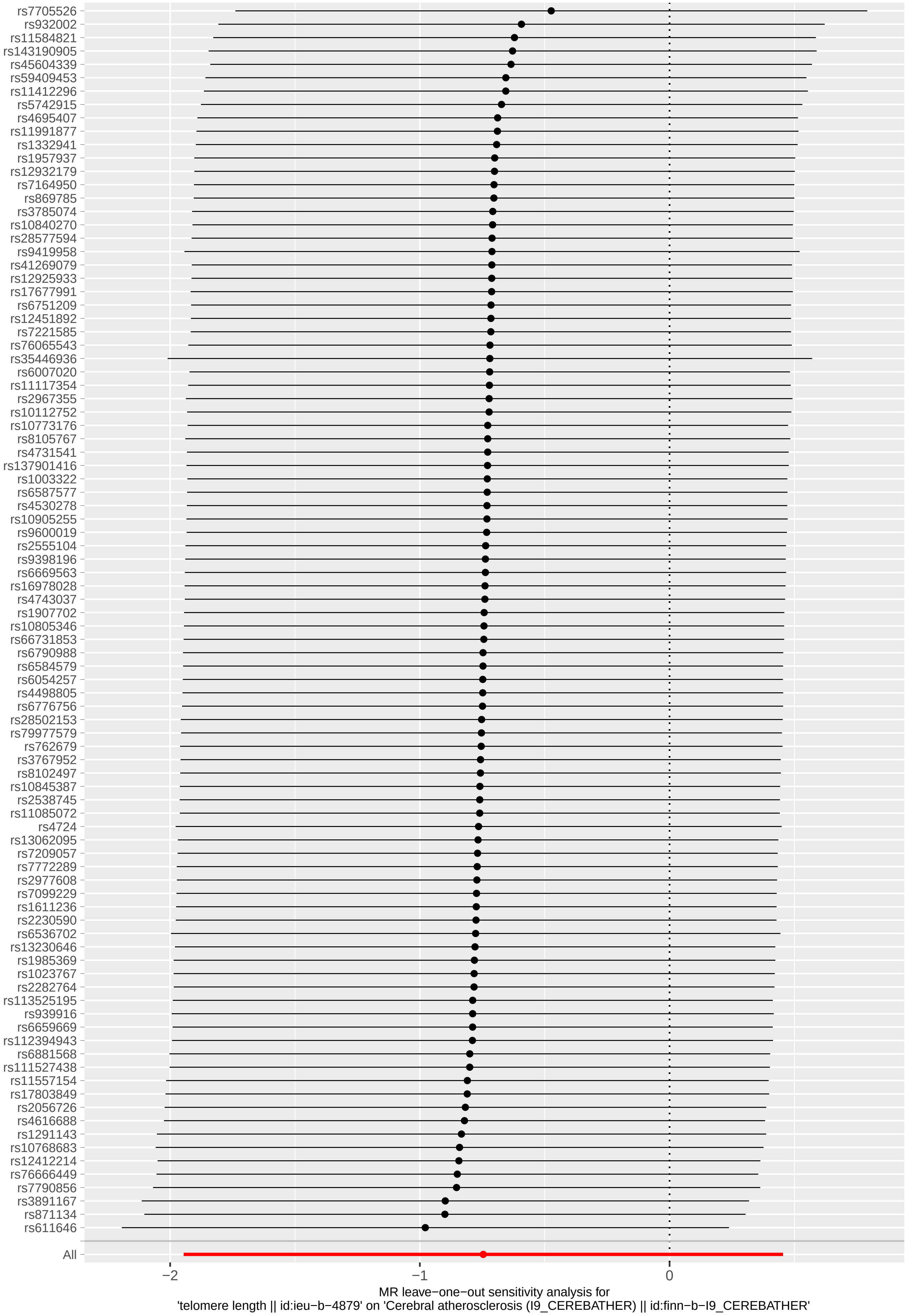

rs7705526
rs932002
rs11584821
rs143190905
rs45604339
rs59409453
rs11412296
rs5742915
rs4695407
rs11991877
rs1332941
rs1957937
rs12932179
rs7164950
rs869785
rs3785074
rs10840270
rs28577594
rs9419958
rs41269079
rs12925933
rs17677991
rs6751209
rs12451892
rs7221585
rs76065543
rs35446936
rs6007020
rs11117354
rs2967355
rs10112752
rs10773176
rs8105767
rs4731541
rs137901416
rs1003322
rs6587577
rs4530278
rs10905255
rs9600019
rs2555104
rs9398196
rs6669563
rs16978028
rs4743037
rs1907702
rs10805346
rs66731853
rs6790988
rs6584579
rs6054257
rs4498805
rs6776756
rs28502153
rs79977579
rs762679
rs3767952
rs8102497
rs10845387
rs2538745
rs11085072
rs4724
rs13062095
rs7209057
rs7772289
rs2977608
rs7099229
rs1611236
rs2230590
rs6536702
rs13230646
rs1985369
rs1023767
rs2282764
rs113525195
rs939916
rs6659669
rs112394943
rs6881568
rs111527438
rs11557154
rs17803849
rs2056726
rs4616688
rs1291143
rs10768683
rs12412214
rs76666449
rs7790856
rs3891167
rs871134
rs611646
All
−2
−1
0
MR leave−one−out sensitivity analysis for
'telomere length || id:ieu−b−4879' on 'Cerebral atherosclerosis (I9_CEREBATHER) || id:finn−b−I9_CEREBATHER'

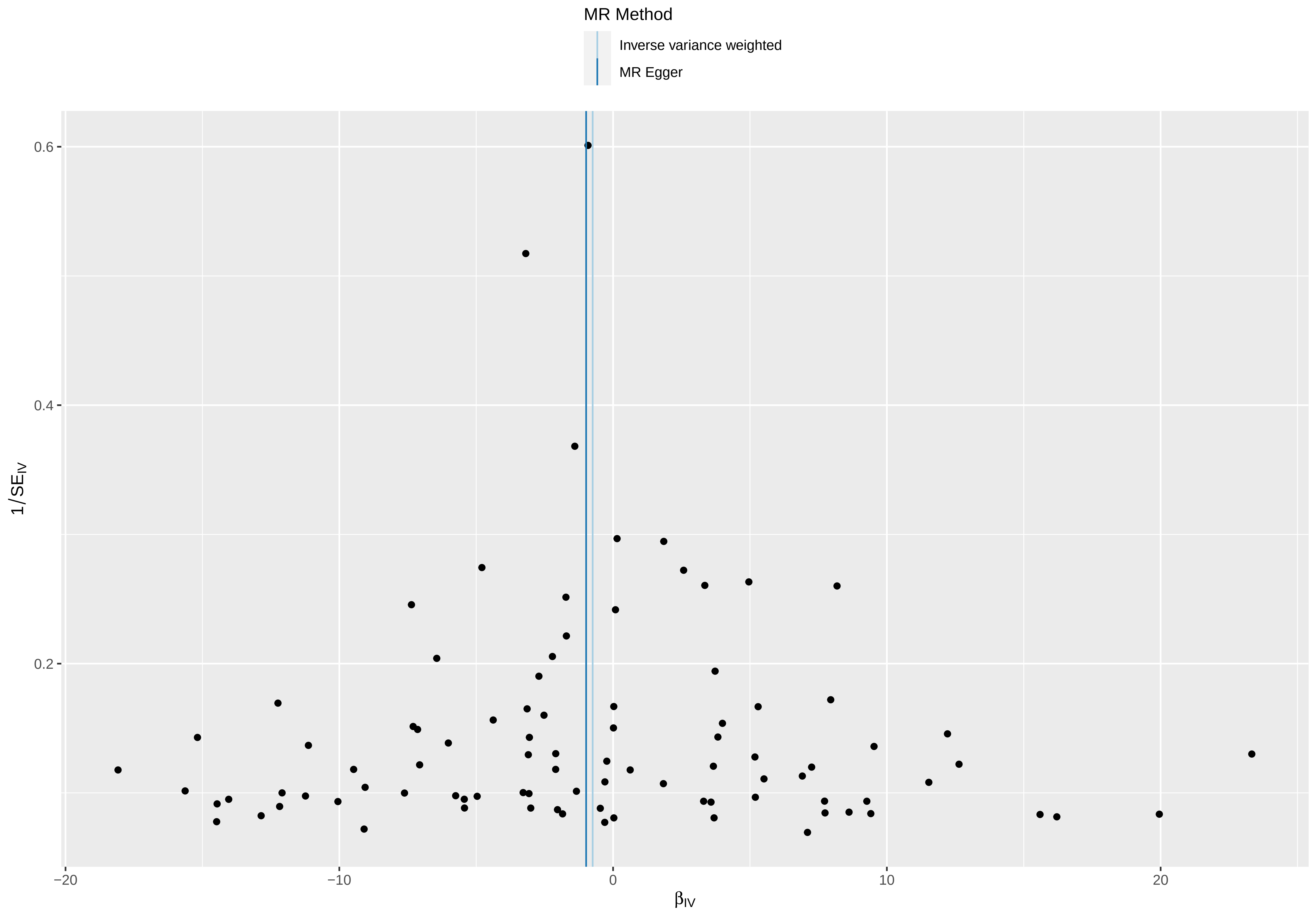

MR Method
Inverse variance weighted
MR Egger
0.6
0.4
0.2
1/SE_IV
−20
−10
0
10
20
β_IV

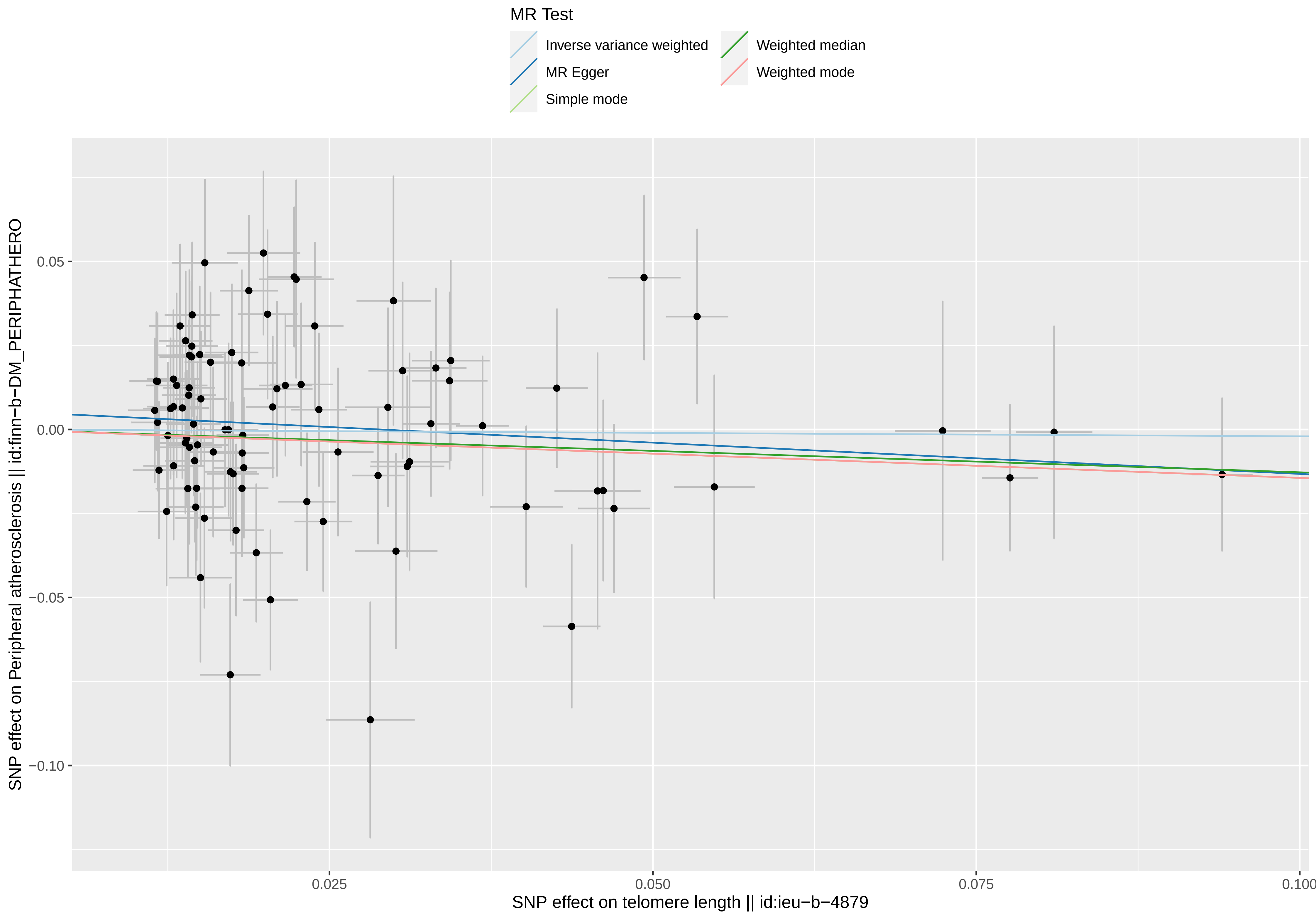

MR Test
Inverse variance weighted
MR Egger
Simple mode
Weighted median
Weighted mode
0.05
0.00
−0.05
−0.10
0.025
0.050
0.075
0.100
SNP effect on Peripheral atherosclerosis || id:finn−b−DM_PERIPHATHERO
SNP effect on telomere length || id:ieu−b−4879

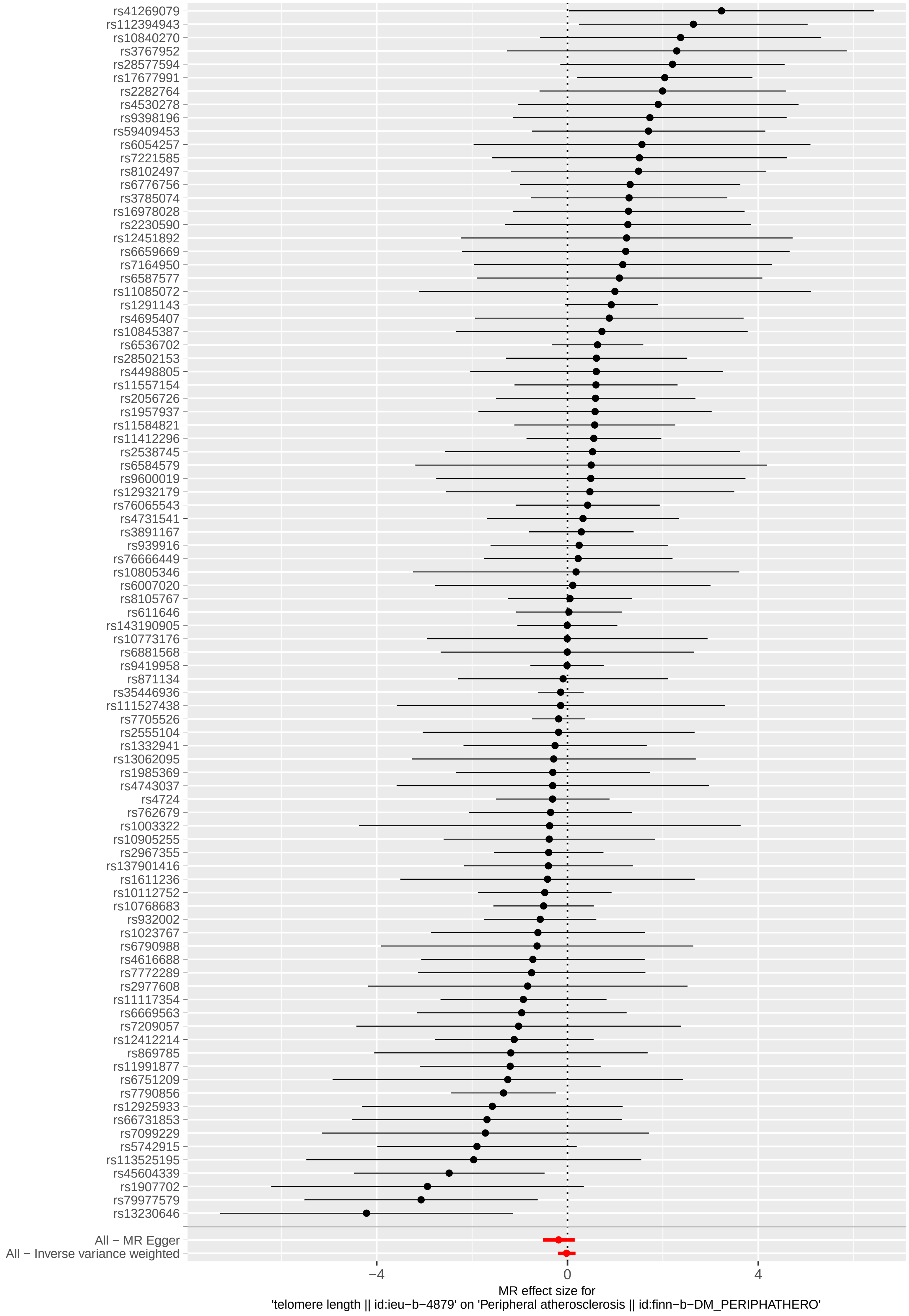
rs41269079
rs112394943
rs10840270
rs3767952
rs28577594
rs17677991
rs2282764
rs4530278
rs9398196
rs59409453
rs6054257
rs7221585
rs8102497
rs6776756
rs3785074
rs16978028
rs2230590
rs12451892
rs6659669
rs7164950
rs6587577
rs11085072
rs1291143
rs4695407
rs10845387
rs6536702
rs28502153
rs4498805
rs11557154
rs2056726
rs1957937
rs11584821
rs11412296
rs2538745
rs6584579
rs9600019
rs12932179
rs76065543
rs4731541
rs3891167
rs939916
rs76666449
rs10805346
rs6007020
rs8105767
rs611646
rs143190905
rs10773176
rs6881568
rs9419958
rs871134
rs35446936
rs111527438
rs7705526
rs2555104
rs1332941
rs13062095
rs1985369
rs4743037
rs4724
rs762679
rs1003322
rs10905255
rs2967355
rs137901416
rs1611236
rs10112752
rs10768683
rs932002
rs1023767
rs6790988
rs4616688
rs7772289
rs2977608
rs11117354
rs6669563
rs7209057
rs12412214
rs869785
rs11991877
rs6751209
rs7790856
rs12925933
rs66731853
rs7099229
rs5742915
rs113525195
rs45604339
rs1907702
rs79977579
rs13230646
All − MR Egger
All − Inverse variance weighted
−4
0
4
MR effect size for
'telomere length || id:ieu−b−4879' on 'Peripheral atherosclerosis || id:finn−b−DM_PERIPHATHERO'

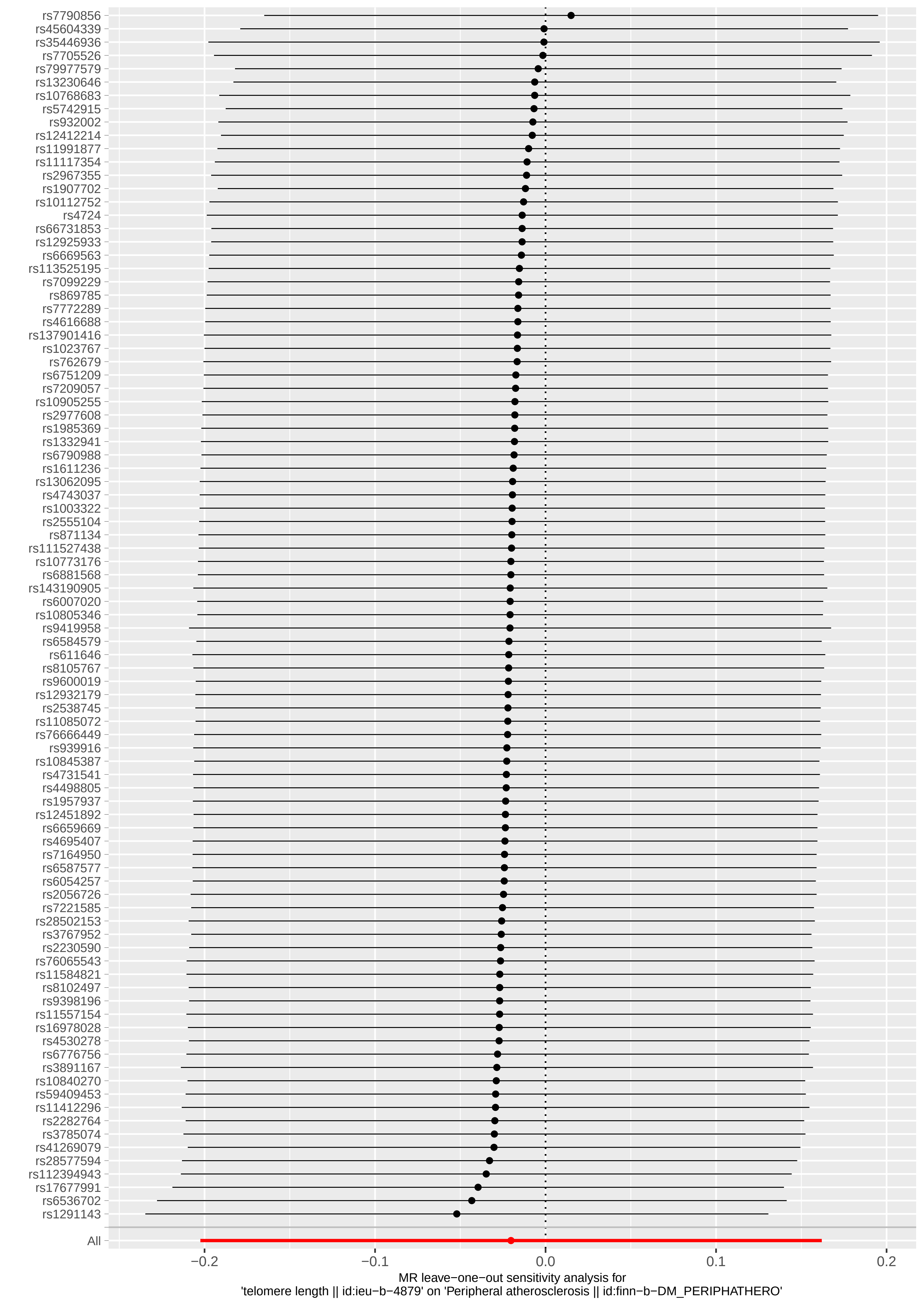

rs7790856
rs45604339
rs35446936
rs7705526
rs79977579
rs13230646
rs10768683
rs5742915
rs932002
rs12412214
rs11991877
rs11117354
rs2967355
rs1907702
rs10112752
rs4724
rs66731853
rs12925933
rs6669563
rs113525195
rs7099229
rs869785
rs7772289
rs4616688
rs137901416
rs1023767
rs762679
rs6751209
rs7209057
rs10905255
rs2977608
rs1985369
rs1332941
rs6790988
rs1611236
rs13062095
rs4743037
rs1003322
rs2555104
rs871134
rs111527438
rs10773176
rs6881568
rs143190905
rs6007020
rs10805346
rs9419958
rs6584579
rs611646
rs8105767
rs9600019
rs12932179
rs2538745
rs11085072
rs76666449
rs939916
rs10845387
rs4731541
rs4498805
rs1957937
rs12451892
rs6659669
rs4695407
rs7164950
rs6587577
rs6054257
rs2056726
rs7221585
rs28502153
rs3767952
rs2230590
rs76065543
rs11584821
rs8102497
rs9398196
rs11557154
rs16978028
rs4530278
rs6776756
rs3891167
rs10840270
rs59409453
rs11412296
rs2282764
rs3785074
rs41269079
rs28577594
rs112394943
rs17677991
rs6536702
rs1291143
All
−0.2
−0.1
0.0
0.1
0.2
MR leave−one−out sensitivity analysis for
'telomere length || id:ieu−b−4879' on 'Peripheral atherosclerosis || id:finn−b−DM_PERIPHATHERO'

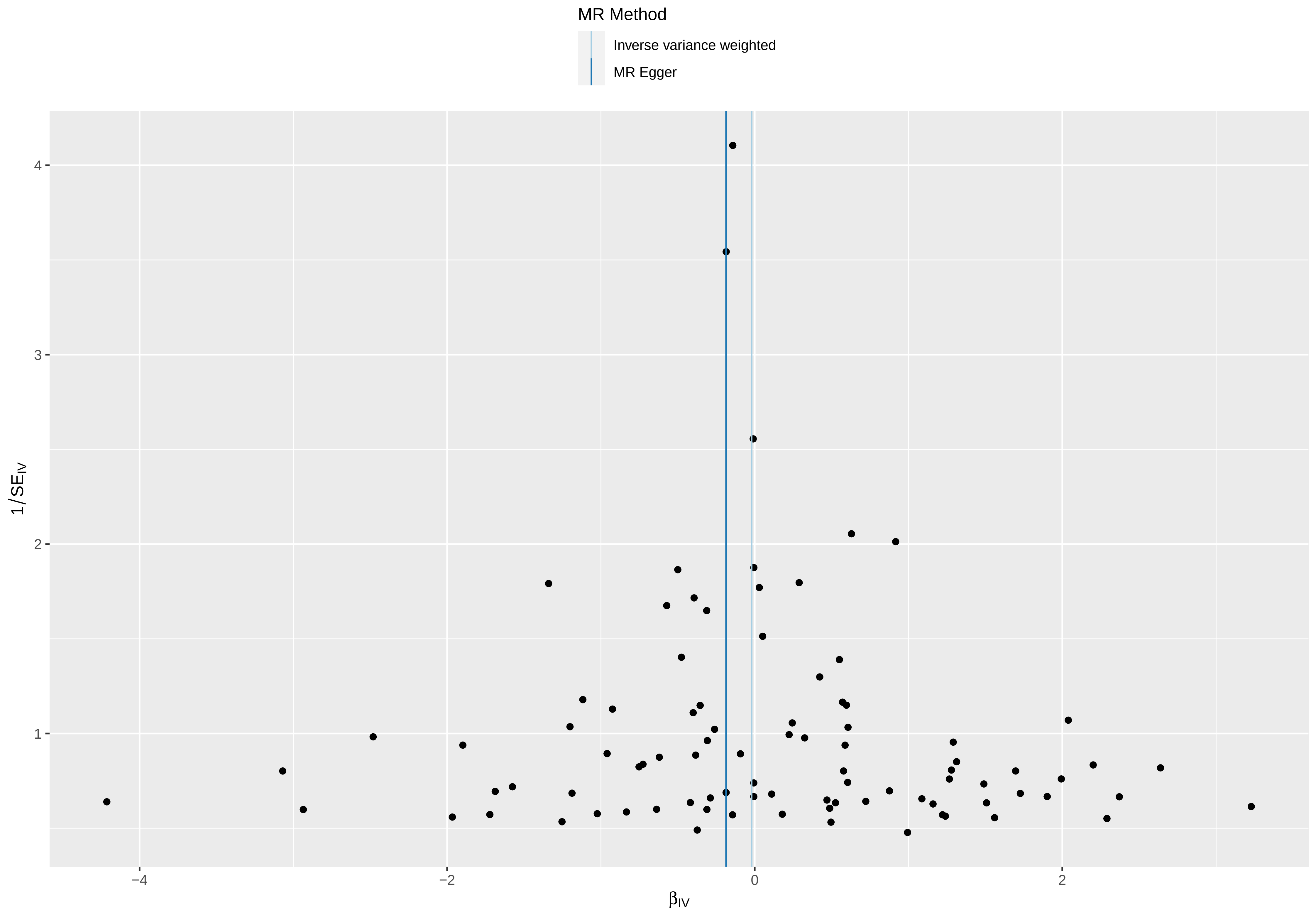

MR Method
Inverse variance weighted
MR Egger
4
3
2
1
1/SE$_{IV}$
−4
−2
0
2
β$_{IV}$

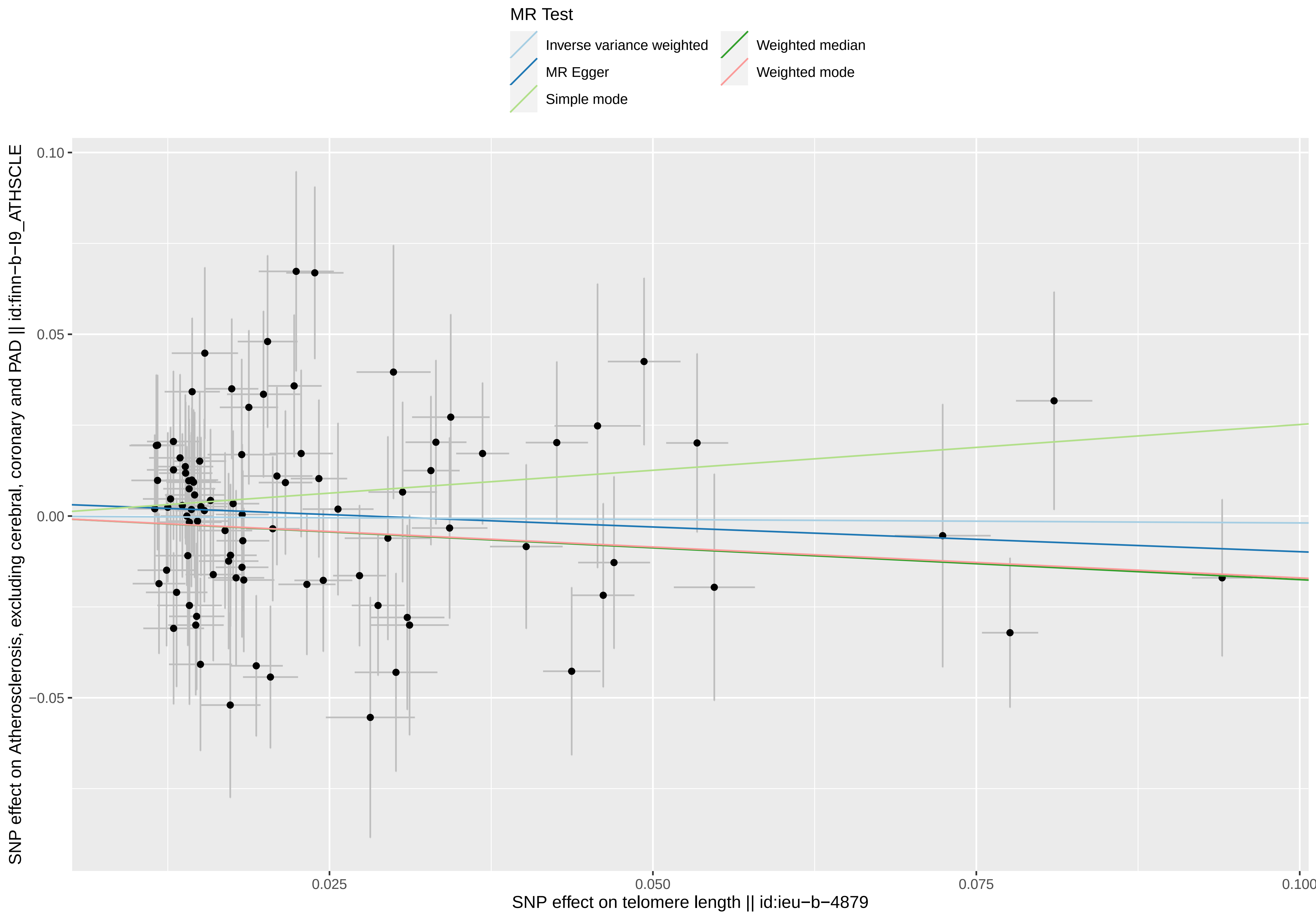

MR Test
Inverse variance weighted
MR Egger
Simple mode
Weighted median
Weighted mode
0.10
0.05
0.00
−0.05
0.025
0.050
0.075
0.100
SNP effect on telomere length || id:ieu−b−4879
SNP effect on Atherosclerosis, excluding cerebral, coronary and PAD || id:finn−b−I9_ATHSCLE

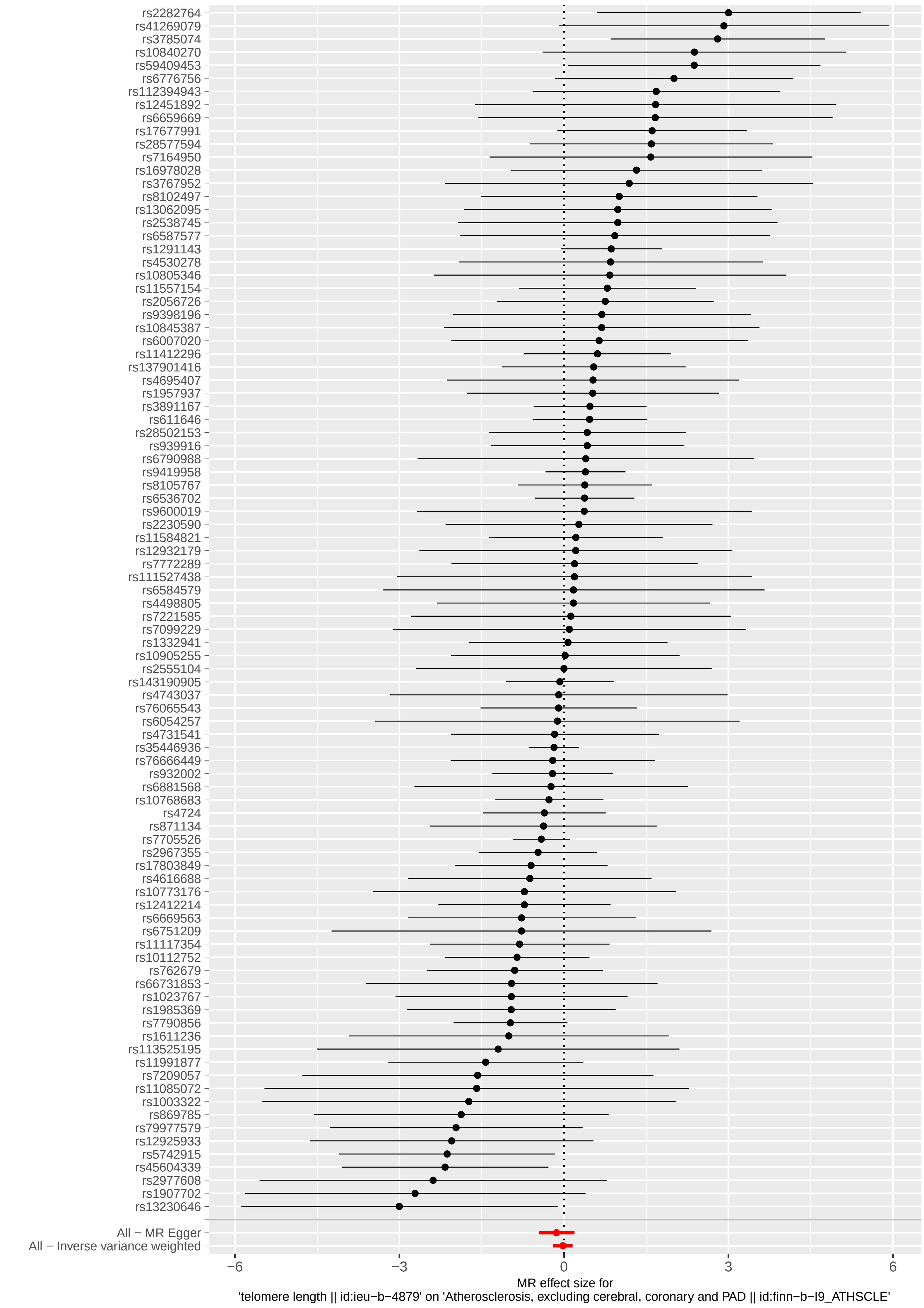
rs2282764
rs41269079
rs3785074
rs10840270
rs59409453
rs6776756
rs112394943
rs12451892
rs6659669
rs17677991
rs28577594
rs7164950
rs16978028
rs3767952
rs8102497
rs13062095
rs2538745
rs6587577
rs1291143
rs4530278
rs10805346
rs11557154
rs2056726
rs9398196
rs10845387
rs6007020
rs11412296
rs137901416
rs4695407
rs1957937
rs3891167
rs611646
rs28502153
rs939916
rs6790988
rs9419958
rs8105767
rs6536702
rs9600019
rs2230590
rs11584821
rs12932179
rs7772289
rs111527438
rs6584579
rs4498805
rs7221585
rs7099229
rs1332941
rs10905255
rs2555104
rs143190905
rs4743037
rs76065543
rs6054257
rs4731541
rs35446936
rs76666449
rs932002
rs6881568
rs10768683
rs4724
rs871134
rs7705526
rs2967355
rs17803849
rs4616688
rs10773176
rs12412214
rs6669563
rs6751209
rs11117354
rs10112752
rs762679
rs66731853
rs1023767
rs1985369
rs7790856
rs1611236
rs113525195
rs11991877
rs7209057
rs11085072
rs1003322
rs869785
rs79977579
rs12925933
rs5742915
rs45604339
rs2977608
rs1907702
rs13230646
All − MR Egger
All − Inverse variance weighted
−6
−3
0
3
6
MR effect size for
'telomere length || id:ieu−b−4879' on 'Atherosclerosis, excluding cerebral, coronary and PAD || id:finn−b−I9_ATHSCLE'

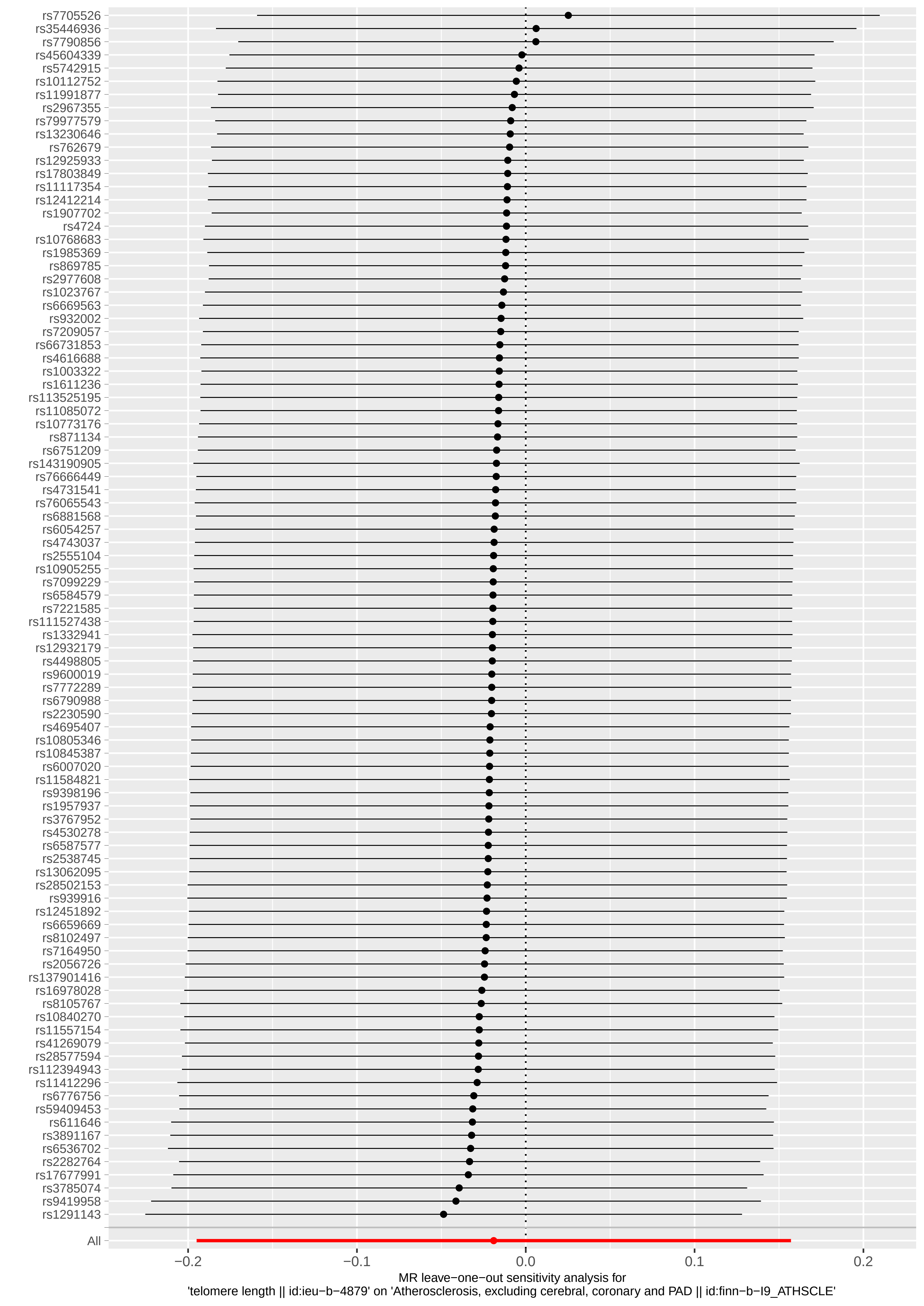
rs7705526
rs35446936
rs7790856
rs45604339
rs5742915
rs10112752
rs11991877
rs2967355
rs79977579
rs13230646
rs762679
rs12925933
rs17803849
rs11117354
rs12412214
rs1907702
rs4724
rs10768683
rs1985369
rs869785
rs2977608
rs1023767
rs6669563
rs932002
rs7209057
rs66731853
rs4616688
rs1003322
rs1611236
rs113525195
rs11085072
rs10773176
rs871134
rs6751209
rs143190905
rs76666449
rs4731541
rs76065543
rs6881568
rs6054257
rs4743037
rs2555104
rs10905255
rs7099229
rs6584579
rs7221585
rs111527438
rs1332941
rs12932179
rs4498805
rs9600019
rs7772289
rs6790988
rs2230590
rs4695407
rs10805346
rs10845387
rs6007020
rs11584821
rs9398196
rs1957937
rs3767952
rs4530278
rs6587577
rs2538745
rs13062095
rs28502153
rs939916
rs12451892
rs6659669
rs8102497
rs7164950
rs2056726
rs137901416
rs16978028
rs8105767
rs10840270
rs11557154
rs41269079
rs28577594
rs112394943
rs11412296
rs6776756
rs59409453
rs611646
rs3891167
rs6536702
rs2282764
rs17677991
rs3785074
rs9419958
rs1291143
All
−0.2
−0.1
0.0
0.1
0.2
MR leave−one−out sensitivity analysis for
'telomere length || id:ieu−b−4879' on 'Atherosclerosis, excluding cerebral, coronary and PAD || id:finn−b−I9_ATHSCLE'

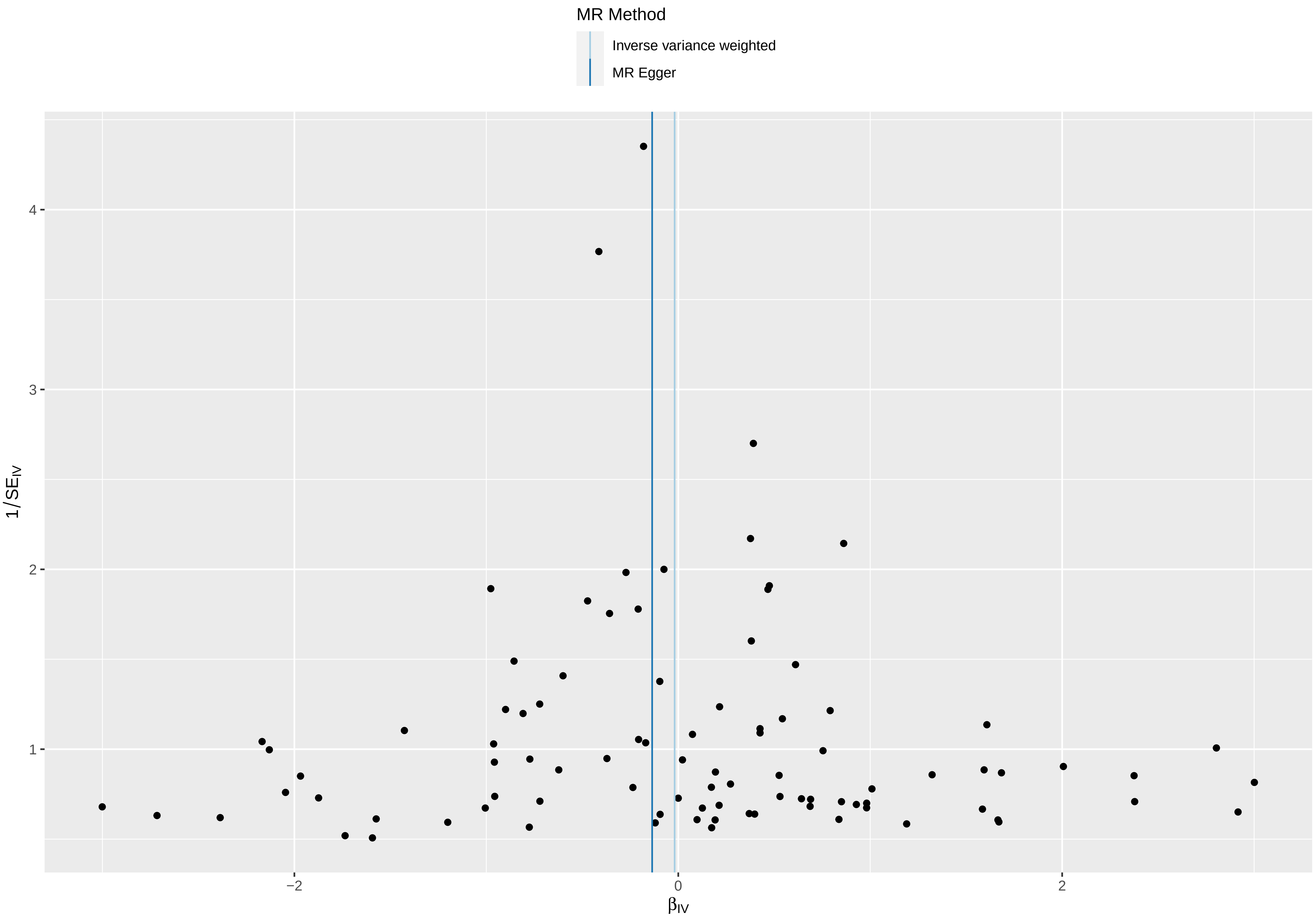

MR Method
Inverse variance weighted
MR Egger
4
3
2
1
1/SE_IV
−2
0
2
β_IV
